# Unravelling miRNA regulation in yield of rice (*Oryza sativa*) based on differential network model

**DOI:** 10.1038/s41598-018-26438-w

**Published:** 2018-05-31

**Authors:** Jihong Hu, Tao Zeng, Qiongmei Xia, Qian Qian, Congdang Yang, Yi Ding, Luonan Chen, Wen Wang

**Affiliations:** 1 State Key Laboratory of Genetic Resources and Evolution, Kunming Institute of Zoology, Chinese Academy of Sciences, Kunming, 650223 China; 2 State Key Laboratory of Hybrid rice, College of Life Sciences, Wuhan University, Wuhan, 430072 China; 3 Key Laboratory of Systems Biology, Innovation Center for Cell Signaling Network, Institute of Biochemistry and Cell Biology, Shanghai Institutes for Biological Sciences, Chinese Academy of Sciences, Shanghai, 200031 China; 4 Institute of Food Crop of Yunan Academy of Agricultural Sciences, Longtou Street, North Suburb, Kunming, 650205 China; 5 Center for Ecological and Environmental Sciences, Northwestern Polytechnical University, Xi’an, 710072 China

## Abstract

Rice (*Oryza sativa* L.) is one of the essential staple food crops and tillering, panicle branching and grain filling are three important traits determining the grain yield. Although miRNAs have been reported being regulating yield, no study has systematically investigated how miRNAs differentially function in high and low yield rice, in particular at a network level. This abundance of data from high-throughput sequencing provides an effective solution for systematic identification of regulatory miRNAs using developed algorithms in plants. We here present a novel algorithm, Gene Co-expression Network differential edge-like transformation (GRN-DET), which can identify key regulatory miRNAs in plant development. Based on the small RNA and RNA-seq data, miRNA-gene-TF co-regulation networks were constructed for yield of rice. Using GRN-DET, the key regulatory miRNAs for rice yield were characterized by the differential expression variances of miRNAs and co-variances of miRNA-mRNA, including osa-miR171 and osa-miR1432. Phytohormone cross-talks (auxin and brassinosteroid) were also revealed by these co-expression networks for the yield of rice.

## Introduction

MicroRNAs (miRNAs) are approximately 21 nucleotides small non-coding RNAs that regulate gene expression at the post-transcriptional level^1^. In plants, miRNAs are transcribed by pol II enzyme and the mature miRNA enters the RNA-induced silencing complex (RISC) and negatively regulates gene expression via perfect or near-perfect sequences complementary with their target mRNA resulting mRNA cleavage or inhibiting mRNA translation^2^. The basic biological function of miRNAs is that they interact with target mRNAs to interfere with the expression level of mRNAs, which can be encoding proteins or factors that control developmental and physiological process in plants and animals^3,4^. Due to the master modulators of gene expression, microRNAs (miRNAs) and their target genes can be exploited for improving agronomic traits in crops^5^.

As a key compontent of the gene regulatory networks, miRNAs have attracted increasing attention with respect to the mechanisms of miRNA-mediated gene regulation^6^. Transcription factors (TFs) are also paramount regulators of gene expression in plants, and thus, the triple-network among miRNA, target-genes and TFs (e.g. miRNA-gene-TF co-regulation network) may conceivably be an important system in regulating plant development. Interaction networks between miRNAs, target genes and TFs are critical for an appropriate balance of gene expression in plants. Many studies integrated the miRNA-mRNA expression profile data for regulatory networks using miRNA target prediction. And the overlapped genes list of these targeted genes of differentially expressed miRNAs and differentially expressed genes in RNA-seq, as well as the mRNA-miRNA pairs exhibited opposite expression profiles, both provided important clues for plant development^7,8^. It is well known that miRNA mainly negatively regulates the expression of its target genes^1^. However, in some cases, the targets of a miRNA are not negatively correlated at the expression level^9,10^, suggesting that miRNA regulation in plant development may be a dynamics process and involve many other factors.

Rice (*Oryza sativa* L.) is one of the essential staple food crops in the world, and improving the yield has been the focus of rice breeding programs. Tillering, panicle branching and grain filling are important traits determining the grain yield. It has been reported that OsmiR156 regulates the famous yield gene *OsSPL14*^11^ and over-expression of OsmiR397 can promote panicle branching, enhancing the grain yield^12^. And miRNAs have also been reported in different tissues or at a certain development stage of rice yield using small RNA sequencing^9,10,13–16^. However, regulation network of these miRNAs with target genes and the signal pathways cross-talk for yield of rice is still unknown. Both osa-miR397 and osa-miR396 invovled in regulating the brassinosteroid (BR) signalling to control grain size and affect yield in rice, their relationship is elusive^5,12,17^. Thus, extensive studies are needed for systematically elucidating the regulation networks in yield of rice, which will provide useful strategies for crop improvement.

Recently, the abundance of data from high-throughput sequencing has greatly facilitated plants research and makes to analyze the gene regulation on systemic level possible. And new challenges arise to effectively integrate the different omics data for studying the biological complexity of yield in rice. Mathematical models of biological systems by integrating experimental and theoretical techniques are required to unravel the complexity of gene regulation in the complex processes^18^. Gene Regulatory Networks (GRNs) is aim to infer complex networks representing transcriptional regulatory relationships from gene expression profiles, such as RNA-seq data^19,20^. Using co-expression network analysis, thousands of genes/transcripts of special interest (e.g. differentially expressed) are utilized to construct the network, identifying key regulators/targets^21^. Using publicly available data and protein-protein interactions (PPIs), a Gene Co-expression Network (GCN) can be constructed on individual sample for candidate gene or regulators selection and improving understanding of regulatory pathways. Although existing non-linearity in GRN, the linear correlation would be more effectively to approximate the network when the samples are not large, especially combining with prior-known background network. However, the linear correlation even might be less practical when the samples are few^22^. Thus, we propose novel edge-like correlations of gene-pairs even in one sample rather than original expressions of genes to reconstruct the GCN. Such correlation-based calculation would usually obtain undirected association, so that, as widely applied in integrative study^23^, in this work, the prior-known miRNA-> Target gene, TF-> miRNA and TF-> gene information are combined with GCN analysis, which come from the well-established public interaction database. Therefore, our GCN can supply potential (directed) regulatory relationships consistent with prior-knowledge in a regulatory network screening manner.

The relations between miRNA-target genes enable users to derive co-expressed genes that may be involved in similar biological processes and functions in plants, similar to previous hypothesis and study in human^24^. The target genes of miRNAs may be co-expression when they are regulated by multiple miRNAs^25^. Using these co-expressed genes, we can theoretically reconstruct the GCNs related to plant development. In contrast to widely used analysis of differential expression in traditional studies, such dynamic regulations (i.e. sample-specific regulation or network) among different individuals (samples) can be characterized by the differential expression variances of miRNAs and co-variances of miRNAs-mRNAs, which would also be important phenotype-related and dynamics-related features in biological processes^26^. In the present study, we develop a new algorithm, called differential edge-like transformation (DET), to analyze the gene regulatory networks and identify key regulatory miRNA in plant development.

## Results

### Gene regulatory network based on DET

**Figure 1:**
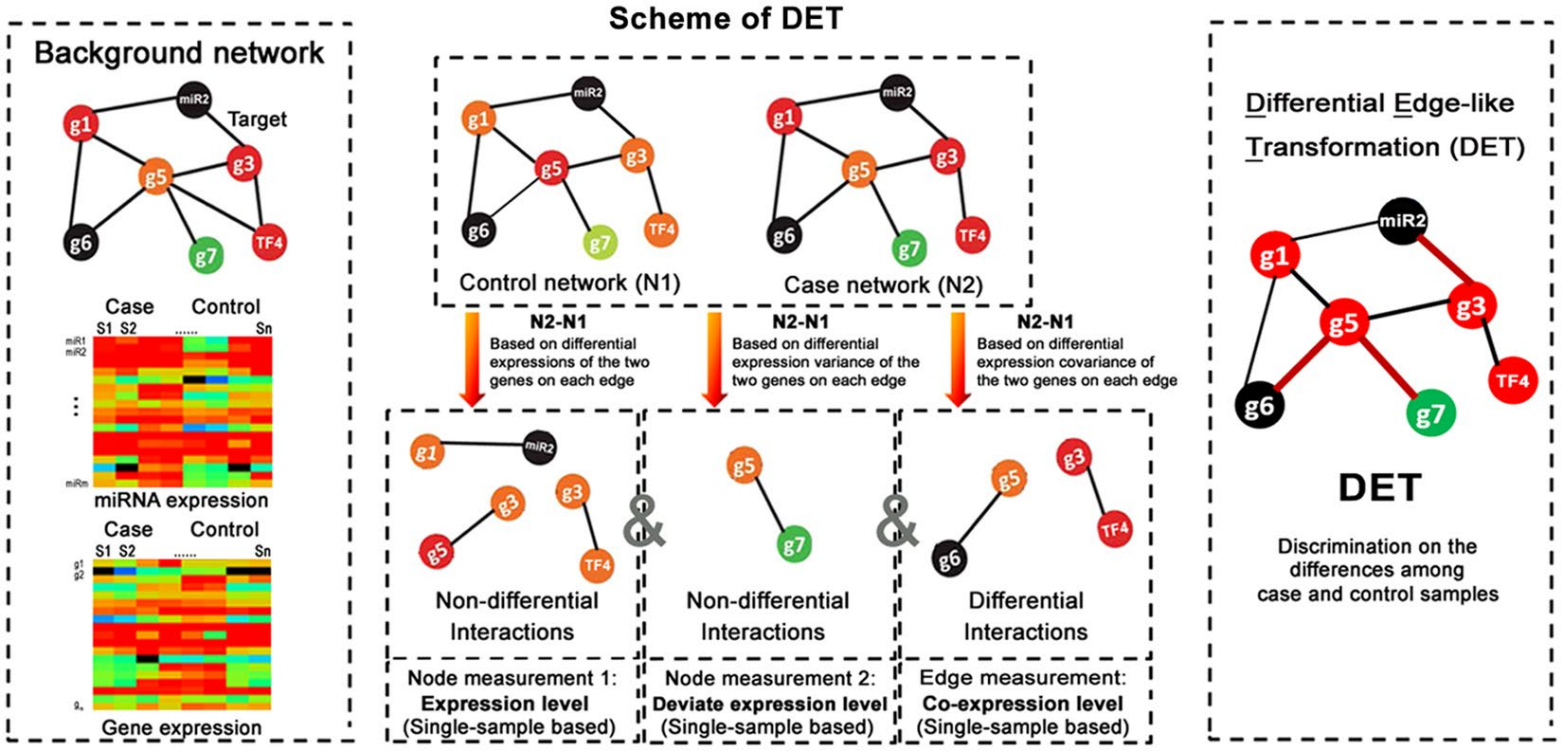
Identification of miRNAs invovled in rice yield using DET method. Workflow of the differential network model based on Differential Edge-like Transformation (DET).

DET transforms original expressions of genes to the edge-like correlations of gene-pairs even in one sample (Fig. 1 and Supplemental Methods). From the statistic viewpoint, those edge-like correlations follow a correlated product distribution (Supplemental Methods), which can be used for significance test of gene-pair associations. This method can be used to not only characterize a single sample by its network according to the weights of edges (i.e. the edge-like correlations or gene-pair associations), but also estimate the dys-regulations of genes in one pair of samples according to their network changes (e.g. the topological differences between networks from control and case samples), thus opening a new way to study the molecular mechanism (e.g. regulatory miRNAs of rice yield) at a network level even with one sample. Using this novel differential network model based on DET, in the present study, the sample-specific miRNAs (SmiRNAs with sample-specific network structures rather than sample-specific expression) and their regulation networks for different tissues or samples were identified.

Although conventional co-expression analysis, e.g. WGCNA (Weight Gene Co-Expression Network Analysis)^22^, can be applied to study co-expression networks, those methods require many samples (N ≥ 10) and thus cannot be applied to the network or miRNA-mRNA interaction analysis with fewer or even two samples (Table 1). In many plant studies, the samples in the data are usually from different tissues or developmental stages, but there are only a few samples for each tissue/stage and thus the traditional network analyses failed.

**Table 1:** Comparison of GRN-DET and WGCNA methods.

Co-expression network	GRN-DET	WGCNA
Model	HMM	Hierarchical clustering
Samples (N≥)	1	10
Gene expression	Yes	Yes
Pearson correlation coefficients	Yes	Yes
Expression variance	Yes	No
Expression covariance	Yes	No
Network changes	Yes	No
miRNA-mRNA interaction	Yes	No

Based on the theory of DET, a new differential network model by combining the miRNA-mRNA regulatory network and its edge-like correlations (Gene regulatory network with DET, GRN-DET) is proposed in this study (Fig. 1). In one condition or in one tissue, there is usually only one sample, we can apply DET to capture the significant gene expression and correlation changes simultaneously in a dynamical and network manner.

### Single sample analysis dependent on the statistics of edge-like correlations

From a viewpoint of statistics, the random variable, edge-like correlation Z (∈ [−∞, ∞]), can be described as the product of two random variables X and Y

1 $${\rm{Z}}={\rm{XY}}=\frac{x-{\mu }_{{\rm{x}}}}{{\sigma }_{{\rm{x}}}}\cdot \frac{y-{\mu }_{{\rm{y}}}}{{\sigma }_{{\rm{y}}}}$$

which follows a correlated product distribution of X and Y, i.e., a product distribution with correlation between X and Y. In particular, given X and Y are statistically independent, the edge-like correlation Z = XY follows a product distribution, e.g., the probability density function of Z as:

2 $${f}_{{\rm{Z}}}({\rm{z}})={\int }_{-\infty }^{\infty }{f}_{{\rm{X}}}({\rm{x}}){f}_{{\rm{Y}}}({\rm{z}}/{\rm{x}})\frac{1}{|{\rm{x}}|}d{\rm{x}}$$

**Figure 2:**
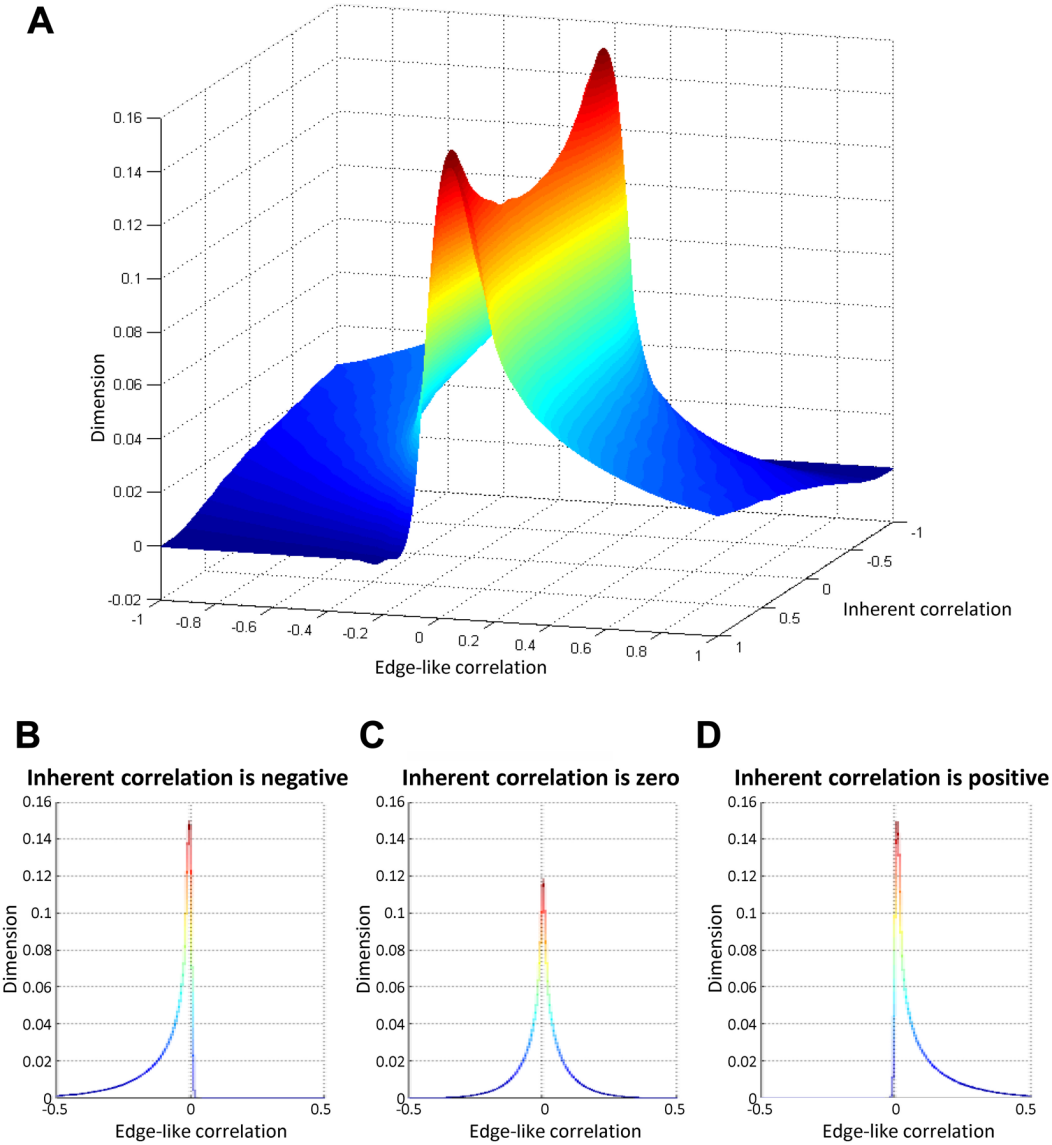
The numerical simulation of the correlated product distribution of edge-like correlation. (**A**) The distribution of edge-like correlation with given inherent correlations. (**B**) The distribution of edge-like correlation with negative-correlation condition. (**C**) The distribution of edge-like correlation with independent condition. (**D**) The distribution of edge-like correlation with positive-correlation condition.

Given X and Y are independent normal distributions (e.g. X and Y are multivariate normal distribution with covariance as 0), Z is normal product distribution; and even when X and Y are statistically dependent (e.g. X and Y are multivariate normal distribution with covariance closed to 1 or −1), Z will be long-tail normal product distribution (Fig. 2).

Furthermore, when X and Y are standard normal distributions, the expectation of Z is just the Pearson’s correlation coefficient ρ_X,Y_ between X and Y, i.e.

3 $${{\rm{\rho }}}_{{\rm{X}},{\rm{Y}}}=\frac{{\rm{E}}[({\rm{X}}-{{\rm{\mu }}}_{{\rm{X}}})({\rm{Y}}-{{\rm{\mu }}}_{{\rm{Y}}})]}{{{\rm{\sigma }}}_{{\rm{X}}}{{\rm{\sigma }}}_{{\rm{Y}}}}={\rm{E}}({\rm{XY}})={\rm{E}}({\rm{Z}})$$

To mimic such correlated product distributions, our numerical simulation is carried on by using matlab function ‘mvnrnd’ to produce multivariate normal distribution X and Y, where the parameter covariance is controlled by the inherentcorrelation between X and Y, i.e. the Pearson’s correlation coefficient between X and Y. Given acovariance matrix, N (N = 20, with 10000 times replication) samples are produced for X and Y, so that, 200000 samples of Z are also be transformed by above DET. Next, the distribution of Z can be estimated by Kernel smoothing function. Finally, the distribution landscape of Z determined by inherent correlation and edge-like correlation can be shown in the 3-D plot (Fig. 2A). Obviously, when the determinant as inherent correlation are from negative correlated (ρ_X,Y_ = −1) to independent (ρ_X,Y_ = 0), and to positive correlated (ρ_X,Y_ = 1), the distribution of edge-like correlation displays left long-tail product distribution (Fig. 2B), and symmetrical product distribution (Fig. 2C), and right long-tail product distribution(Fig. 2D) respectively. Thus, the Mann–Whitney U test is simply used to evaluate the statistic significance of edge-like correlations in this work.

In addition to above numerical validation on the work of edge-like correlation, the theoretical result of edge-like correlation on a single sample is also supplied in Supplemental Methods.

### Validation of GRN-DET method using public rice grain filling data

**Figure 3:**
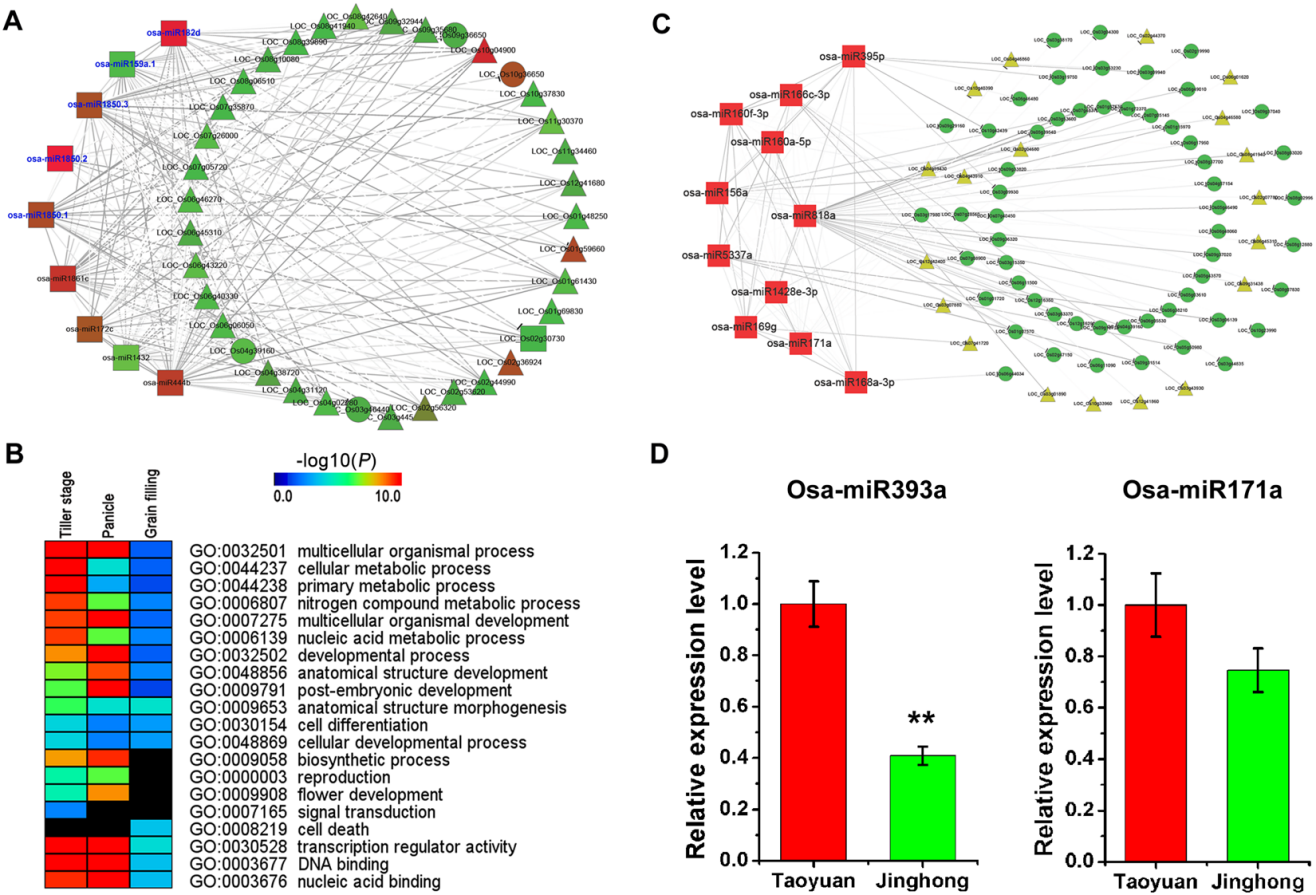
Co-expression networks and GO enrichment of identified candidate yield miRNAs. (**A**) Co-expression networks of identified candidate yield miRNAs with verified yield-associated miRNAs and their regulated genes or TFs in superior and inferior spikelets. (**B**) GO enrichment of the co-regulated genes with SmiRNAs identified by DET in the three key stages (tiller, panicle and grain filling) for rice yield. (**C**) Co-expression networks of identified candidate yield miRNAs with verified yield-associated miRNAs and their regulated genes or TFs in Tao yuan ultra-high yield rice. (**D**) Quantitative real-time PCR (qRT-PCR) validation of osa-miR393a and osa-miR171a in tillers and young panicles at Taoyuan and Jinghong rice, respectively. The significant difference of expression level between Taoyuan and Jinghong in IR64 was determined by Student’s t test, **p < 0.01.

Firstly, we conducted the GRN analyses based on DET using the public small RNA and gene expression dataset from NCBI, which was originally used to investigate rice grain filling. During grain development, poor grain-filling in inferior spikelets greatly decreased the yield of *Oryza sativa* spp. *japonica* cv. ‘Xinfeng 2’^9^. Grains of superior spikelets and inferior spikelets (10 days after flowering (DAF), 15DAF, 21DAF, and 27DAF, respectively) from rice cultivar ‘Xinfeng 2’ were collected and sequenced for small RNA and mRNA profiling by these two previous studies^9,27^. The co-expression analysis of superior spikelets and inferior spikelets showed that 47 differentially expressed miRNAs (DEmiRNAs) might influence grain-filling of rice, and the differential networks as co-variances of these miRNAs with mRNA were also constructed. For example, the expressions of the target genes regulated by osa-miR164 were decreased during the grain-filling. Meanwhile, these expression patterns were significantly different between superior and inferior spikelets. In addition, 20 SmiRNAs related to grain-filling between the two different spikelets were identified by DET (Fig. 3A and Table S1). Most of the SmiRNAs have been reported to be involved in rice grain-filling, including osa-miR444b, osa-miR1861, osa-miR172c, osa-miR1862 and so on^28^ (Fig. 3A). Using GRN-DET, we identified some miRNAs which are no differential expressions but have differential regulations (networks) between superior spikelets and inferior spikelets, including osa-miR395n, osa-miR164 a/b/f, osa-miR2102-5p, osa-miR1432 and osa-miR166k/l (Table S1 and Fig. S1). Our network analysis further showed that osa-miR1861 regulated many genes or TFs in the superior spikelets and had cross-talk with the verified yield-associated osa-miR159a.1 (Fig. 3A). The results were consistent with previous studies, suggesting it could be an important regulator of rice yield^9,13^. GO enrichment analysis showed that the genes co-regulated by miRNAs identified by DET were involved in ‘nitrogen compound metabolic process’, ‘anatomical structure morphogenesis’, ‘cell differentiation’ and ‘cell death’ (Fig. 3B). These results were consistent with previous studies about metabolic pathways from ‘embryo differentiation’ at the early phase to ‘senescence and dormancy’ at the late filling phase^29^.

Secondly, to validate the identified SmiRNAs by our network analysis based on DET in the grain filling, we also performed a conventional WGCNA study on 438 miRNAs from four published rice grain filling small RNA sequencing data^9,10,13,28^. On the combined 22 samples, WGCNA can be applied to obtain one network (e.g. common associations) for miRNAs across multiple samples, rather than individual samples. A total of 9 co-expression modules (e.g. common co-expression pattern across multiple samples) were obtained from highly correlated gene expression patterns (Fig. S2A,B). Eight miRNAs were both identified in DET network and WGCNA module, including osa-miR169a, osa-miR166l, osa-miR444b, osa-miR1432 and so on (Fig. S2C and Table S1). However, unlike WGCNA, different interactive networks of these miRNAs with other miRNAs or mRNAs were revealed by GRN-DET (Figs 3A and S1). Thus, osa-miR1432 and osa-miR444b might be the key yield-associated miRNA in grain filling of rice (Fig. 3A). Therefore, our method can actually detect sample-specific network and miRNAs (SmiRNAs), and some of them are also interactive on the conventional multi-sample based common network, which support the effectiveness of our method.

### Indentify regulatory miRNAs for yield of rice using GRN-DET on in-house data

Using the effective method of GRN-DET, we can also analyze other important stages for yield of rice. Taoyuan, Yunnan, China, has been a well-known amazing place where the highest rice yield in the world was recorded^30^. We collected the tillers, young panicles and flag leaves from the rice variety IR64 at Taoyuan and the yield control place Jinghong. In order to investigate the miRNA regulation roles for ultra-high yield at Taoyuan, we sequenced small RNAs and mRNAs of tissues from three developmental stages (i.e. tillering, panicle branching and grain filling) of rice planted at Taoyuan and Jinghong, respectively. Totally, 234, 82 and 134 DEmiRNAs were identified between the three cases and their controls, respectively (Table S2). Moreover, using GRN-DET, 40, 35 and 34 SmiRNAs for tillers, panicles and flag leaves were identified, respectively (Table S1). Using the corresponding transcriptome data, differential networks of the SmiRNAs and their regulated genes or TFs derived from our GRN-DET have also shown regulatory differences between Taoyuan and Jinghong rice (Figs 3C and S3, S4). Some of these SmiRNAs were not differential molecules which cannot be measured by traditional method but have differential regulations or networks which were identified by our method, such as osa-miR396b-5p in tillers, osa-miR171a in young panicles and osa-miR812m in flag leaves, respectively (Fig. S3 and Tables S1, S2). At young panicle stage, the osa-miR171a regulated the expression levels of more target genes in Jinghong than that of Taoyuan, which may suppress some genes invovled in panicle development, leading to decrease yield of rice (Fig. S3B). In Taoyuan and Jinghong, the three miRNAs were regulated different targets and had differential networks, affecting the key development tissues for yield in rice (Fig. S3).

Two miRNAs (osa-miR393a and osa-miR171a) were randomly selected to validate the expression level by quantitative RT-PCR (qRT-PCR) in IR64 from tillers and panicles of Taoyuan and Jinghong rice, respectively, and the results are consistent with the sequencing data (Fig. 3D). In other words, the functions of those SmiRNAs are facilitated not at the expression level but at the network level. GO and KEGG enrichment analyses of the genes regulated by these SmiRNAs in Taoyuan rice showed that they involved in flower development, embryonic development, nucleic acid metabolism, nitrogen compound metabolic process and so on (Figs 3B and S5).

**Figure 4:**
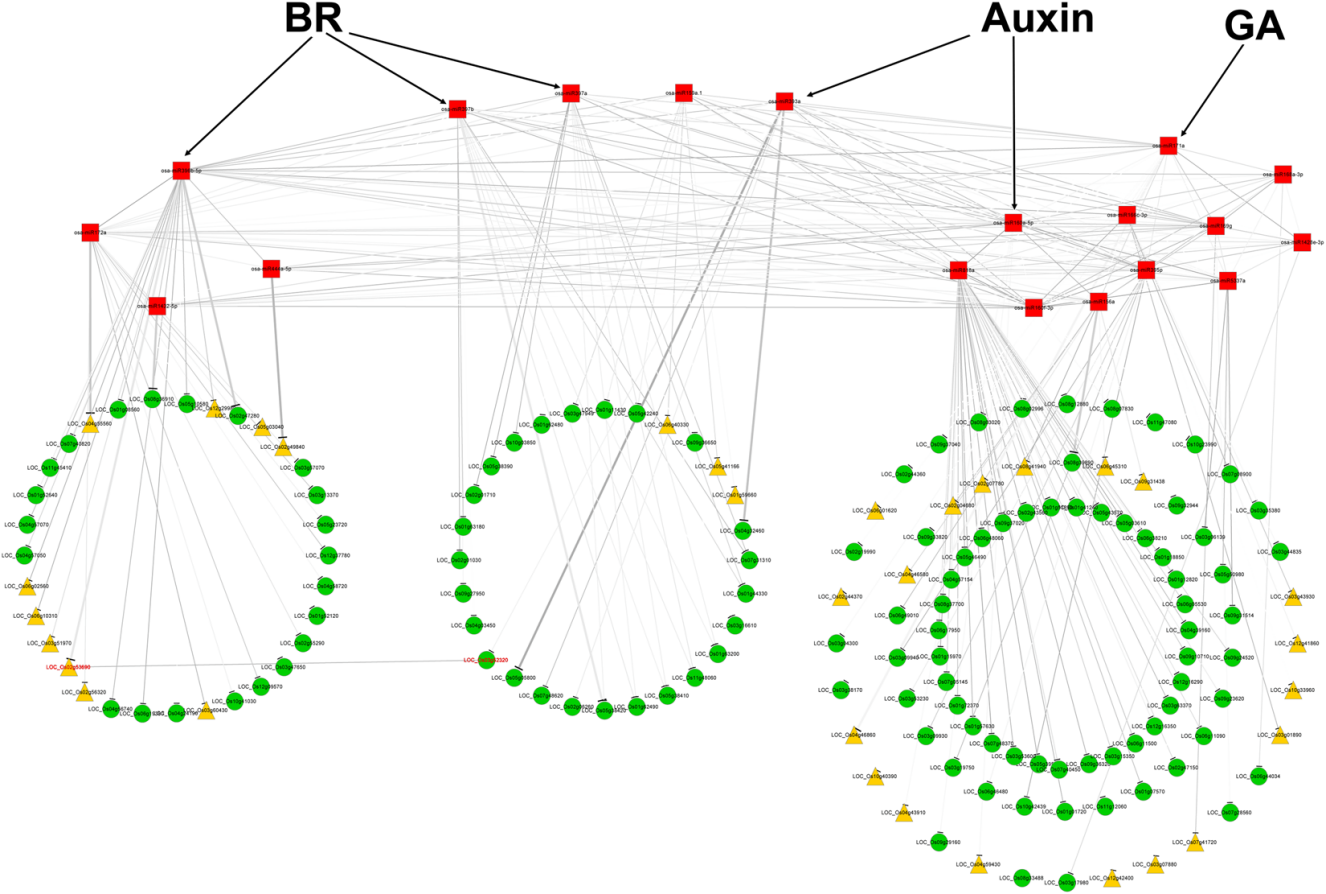
Co-expression networks of candidate yield miRNAs identified by differential edge-like transformation (DET) and reported yield miRNAs with their target genes for high yield in rice from tillering to grain filling stages. The bold line indicated the reported yield miRNAs and their confirmed targets (See Table S4). BR, brassinosteroid, GA, gibberellin.

**Figure 5:**
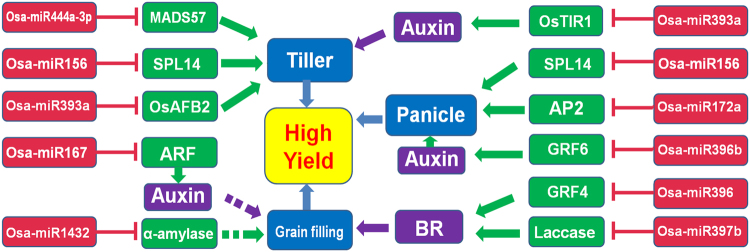
The potential regulatory network model of miRNAs for yield at three stages (tillering, panicle branching and grain filling) in rice. Solid and dashed arrows are the verified and predicted regulatory relationships, respectively.

Plant yield has demonstrated to be control by various plant hormones, including auxin, brassinosteroid, gibberellic and cytokinin^31^. In our study, we found that miRNAs invovled in two phytohormones, auxin and BR signaling pathways to affect rice yield (Figs 4 and 5). Auxin is mainly participate in the growth periods (vegetable and reproductive growth stages), while BR is invovled in grain filling, affecting the grain size (Fig. 5).

## Discussion

MiRNA-mRNA interactions have been predicted by some toolkits or pipelines, including Mtide and Sparta^32,33^. Most of the packages or tools were made for animals or human which have much other information (for instance, validated gene-gene interactions) to integrate for identifying key miRNAs. However, the algorithm for key regulatory miRNA identification in plant development is lacking. Herein, the new algorithm of differential edge-like transformation (DET) was developed for effectively identifying the key regulatory miRNA for rice yield. Although miRNAs have been reported being regulating yield, no study has systematically investigated how miRNAs differentially function in high and low yield rice, in particular at a network level. In this study, based on DET, we construct miRNA differential regulation networks in the three key developmental stages between high and low yield rice, which are further exploited to reveal novel regulatory roles of netted miRNAs in grain yield. The application of DET to the grain-filling and yield of rice datasets demonstractes that GRN-DET provides high accuracy in identifying regulatory miRNAs using RNA-seq and small RNA sequencing.

Using GRN-DET, osa-miR171 and osa-miR1432 has been screened to be involved in panicle branching and grain filling (Figs 3 and S1, S3). Scarecrow (SCR) is a member of GRAS family which is essential for asymmertric cell division of shoot in Arabidopsis^34^. Many members of the osa-miR171 target the SCR transcription factor (Table S3). In tomato, over-expression of the target gene of miR171 (*SlGRAS24*) has been reported to cause alteration of inflorescence architecture and lateral branch number^35^. The target gene of osa-miR1432 is alpha-amylase (LOC_Os08g36910) that is a starch-hydrolyzing enzyme. It’s reported that suppression of α-amylase can ameliorates the grains during the ripening period under high temperature^36^.

Some of the miRNAs showed cross-talks for yield in rice, for example LOC_Os02g53690 (GRF) targeted by osa-miR396 and LOC_Os03g52320 (GRF-interacting factor 1, *GIF*1) which is the target of osa-miR393 (Fig. 4). *GIF* genes have been reported to play important role in cell proliferation and shoot apical meristem, detemining organ size in Arabidopsis^37^. The two miRNAs (osa-miR396 and osa-miR393) are functionally characterized to be involved in tiller and panicle branching, respectively^38,39^. Thus, the cross-talk may provide a clue for better understanding the miRNA regualtion in rice yield.

Phytohormones play vital roles in the plant growth and development, including yield in crops^40^. Auxin has demostrated to regulate stem elongation, lateral branching and vascular development^31^. Different miRNAs (osa-miR393a, osa-miR396b and osa-miR167) with their targets were involved in auxin signaling to affect tillering, panicle branching and grain filling in rice (Fig. 5). The results of our study also provide some insights to the cross-talk and co-expression network of osa-miR397 and osa-miR396 which involved in regulating the BR signalling to control grain size and affect yield in rice (Fig. 4). As the negative regulator of BR signalling, GSK2 interacts with OsGRF4 (target of osa-miR396) and inhibits its transcription activation activity to mediate the specific regulation of grain length in rice^17^. And suppressing of osa-miR396 (MIM396) up-regulated many auxin synthesis and response genes (*YUCCA*, *ARFs* and *GH3*), revealing OsGRF6 is a positive regulator of auxin signalling pathway^38^. Overexpression of osa-miR397 also altered lots of the brassinosteroid-related genes^12^. Therefore, we documented here that some key miRNAs via phytohormone (auxin and BR) involved in the regulation of yield in rice from tillering to grain filling stages (Fig. 5). Furthermore, BR can promote GA accumulation by regulating the expression of GA metabolic genes to stimulate cell elongation^41^. SCR (target of miR171) has also been reported to interact with DELLA porteins, mediating the GA-regulated chlorophyll biosynthesis^42^ (Fig. 4). Using GRN-DET, the phytohormone-related miRNAs and targets interactions as well as their networks were revealed (Fig. 4). Thus, the cross-talk of BR and auxin or BR and GA may play important roles in the yield of rice.

Based on the small RNA, RNA-seq and reported verified miRNA-target interaction data, highly reliable and biologically meaningful co-expression networks based on DET have been constructed for better elucidating the regulatory roles of miRNAs in high yield of rice. This work not only identified new regulatory miRNAs affecting the yield of rice, but also provides a method to systematically reveal miRNA regulation networks in limited but key samples. The results also provide clues for future efforts of increasing rice yield using non-coding RNAs.

## Materials and Methods

### Plant materials and high-throughput sequencing

Small RNA and transcriptome sequencing were performed for the variety IR64 in Taoyuan (ultra-high yield) and Jinghong (natural yield) at three different stages (tillering, panicle branching and grain filling) for the yield of rice. Total RNA was extracted from three tissues (tillers, young panicles and flag leaves) of rice IR64 using the Trizol (Invitrogen). Libraries were generated according to the manufacturer’s recommendations and sequenced by Illumina HiSeq2500 platform (Supporting Information). Bioinformatic analysis of small RNA-sequencing and RNA-seq data were as previous studies^2,43^ (Supporting Information). All the small and RNA sequencing data were deposited in the NCBI Short Read Archive (SRA) (http://www.ncbi.nlm.nih.gov/sra) under the accession number: SRP134071 and SRP144409, respectively.

### Data sources

To collect the miRNAs and target genes involved in rice yield, we conducted literature search for studies that directly assessed miRNA regulation in tiller, young panicle, flag leaf and grain filling^13,16,39,44^. Then, a total of 150 yield associated conserved miRNAs were retrieved from miRBase (release 21; http://www.mirbase.org/)^45^. The degradome sequencing data from different tissues in rice (GSM434596, GSM455938, GSM455959 and GSM476257) were also used in this study. The targets of these miRNAs by predicted, degradome sequencing and experimentally verified were merged^46^ (Table S3). In addition, the small RNA and transcriptome sequencing data from superior and inferior spikelets in ‘Xingfeng2’ rice grains reported previously were also collected for methold validation^13,16,27^.

### Gene regulatory networks with DET (GRN-DET)

Generally, the differential co-expression analysis is to see if or not the expression correlation of a gene-pair (e.g. two genes or molecules) changes between control and case samples^22,47^. Thus, the Pearson correlation coefficient (PCC) between genes *i *and *j* in control or case samples can be calculated as:

4 $$\begin{array}{l}{\rm{Correlation}}\,{\rm{in}}\,{\rm{control}}\,{\rm{condition}}:\frac{1}{{{\rm{m}}}_{{\rm{x}}}-1}\sum _{{\rm{k}}=1}^{{{\rm{m}}}_{{\rm{x}}}}(\frac{{x}_{{\rm{ik}}}-{\mu }_{{\rm{xi}}}}{{\sigma }_{{\rm{xi}}}}\cdot \frac{{x}_{{\rm{jk}}}-{\mu }_{{\rm{xj}}}}{{\sigma }_{{\rm{xj}}}})\\ {\rm{Correlation}}\,{\rm{in}}\,{\rm{case}}\,{\rm{condition}}:\frac{1}{{{\rm{m}}}_{{\rm{y}}}-1}\sum _{{\rm{k}}=1}^{{{\rm{m}}}_{{\rm{y}}}}(\frac{{y}_{{\rm{ik}}}-{\mu }_{{\rm{yi}}}}{{\sigma }_{{\rm{yi}}}}\cdot \frac{{{\rm{y}}}_{{\rm{jk}}}-{\mu }_{{\rm{yj}}}}{{\sigma }_{{\rm{yj}}}})\end{array}$$

where, there are m_x_ control samples and m_y_ case samples; for a control sample *k*, its expressions on genes *i* and *j* are *x*_ik_ and *x*_jk_; the expression average and variance for gene *i* (or gene *j*) on control samples are *μ*_xi_ and *σ*_xi_ (or *μ*_xj_ and *σ*_xj_); and conveniently, the sample in case has these similar variables and annotations but with index *y*. Note that a gene can be replaced by a molecule in this mathematical framework.

DET transforms the expression of genes to the edge-like correlation of gene-pairs in one sample, and the mean of edge-like correlation of a gene-pair in all control and case samples is just the Pearson correlation coefficient on all samples, so that this measurement has equivalent numerical meaning for any control or case sample.

For each tissue, the selected miRNAs or mRNAs and their edge-like correlations will consist of a tissue-specific network and displayed in a topological structure, where the strength of each pair of molecules (e.g., molecules *i* and *j*) in the network is the corresponding edge-like correlation (e.g., edge between molecules *i* and *j*). Besides, for any miRNA, its average PCC (i.e. the edge-like correlation) with other relevant miRNAs or mRNAs in control or case is defined as AP_control_ and AP_case_, then a factor as PCC induced key associated score for this miRNA is computed as |AP_case_ − AP_control_|.

### miRNA-TF-gene network analysis and key miRNAs identifying

Based on RiceNetDB^48^ and RiceNet^49^ (version 2), the TF-gene/miRNA regulatory relations were deciphered. To further illustrate the regulatory structure of miRNA-TF-gene, we re-analyzed the topological structures among miRNA-targets, miRNA-TF and TF-gene associations. Known yield-associated miRNAs from literatures were collected (Table S4). The key miRNAs selection was performed by Pearson correlation coefficient (PCC) between each pair of miRNAs or genes and known yield-associated miRNAs calculated based on their edge-like correlation profiles, and the sample-specific miRNAs, (SmiRNAs) (i.e. with highest key-associated score) were found in tillers, panicles, flag leaves and grains, respectively.

### Identified key miRNAs for yield of rice using GRN-DET

To estimate the accuracy of GRN-DET, we have analyzed the small RNA sequencing data for yield in rice. To construct a miRNA-target gene-TF co-regulation network for the trait of rice yield by DET, the GRN was conducted in the following ways (see the detail methods in Supporting Information): (i) a set of 150 reported sequencing-screened yield related miRNAs and target genes from literatures or degradome data were collected, including 12 experimentally verified yield-associated miRNAs (Tables S3 and S4); (ii) DET was used to transform the original gene expression profiles (i.e. gene *v*.*s*. sample data matrix) to edge-like correlation profiles (i.e. gene-pair *v*.*s*. sample data matrix); (iii) the correlation between each pair of miRNAs or mRNAs (target genes or TFs) and 12 verified yield-associated miRNAs on each sample were further obtained based on such edge-like correlation profiles (the correlation of a gene-pair in a sample is significant when its edge-like correlation is large, otherwise non-significant when the edge-like correlation is small, where the difference significance of edge-like correlation in one *v*.*s*. multiple samples can be evaluated by Mann–Whitney U test); (iv) on the edge-like correlation weighted miRNA-target gene-TF co-regulation network, the key regulatory SmiRNAs related to rice yield were identified according to the PCC-induced key-associated scores (Supporting Information).

### GO and KEGG pathway enrichment analysis

According to their degrees of nodes, network hubs were determined and the top 5% of miRNAs, TFs and genes were considered as hub components. Functional classifying the targets of these miRNAs was enriched by AgriGO (Gene Ontology) (http://bioinfo.cau.edu.cn/agriGO/)^50^ and KEGG database (/ftp.genome.jp/pub/kegg/pathway/). A *p*-value with 0.05 as the cutoff for enriched terms or pathways in GO and KEGG.

### Weighted correlation network analysis (WGCNA)

Based on a group of the collected miRNA profiles for grain filling (DAF, day after flowering) in rice^9,10,13,28^, a R package WGCNA^22^ has been carried on and several co-expression modules have been identified. The miRNA expression profilings were included at the three filling stages: milk-ripe (5DAF, 10DAF), soft-dough (12DAF, 17DAF) and hard-dough (21DAF, 27DAF). Using Pearson correlation coefficient, the gene co-expression similarity were identified and clustered into network modules.

### Stem-loop RT-PCR and quantitative real-time PCR

Total RNA was extracted from tillers and young panicles of the variety IR64 in Taoyuan and Jinghong. For each reverse-transcription (RT) reaction, 2 μg of total RNA was reverse transcribed into cDNA using a miRNA specific stem-loop primers and reverse transcriptase (Takara, Dalian, China) as previously described^51^. Reverse transcription was performed with pulsed RT: the reactions were incubated for 30 min at 16 °C, followed by 60 cycles at 30 °C for 30 s, 42 °C for 30 s and 50 °C for 1 s and finally the reactions were terminated at 70 °C for 5 min. Real time qRT-PCR analysis of the miRNA and their targets was performed using the FastStart Universal SYBR Green Master Mix (Roche) on the StepOne plus PCR platform (AppliedBiosystems). U6 snRNA was used as an endogenous control. The primers were listed in Table S5. To avoid non-specific amplification, melting curve was carried out for each PCR product. All qRT-PCR reactions were performed with three biological replicates and the relative gene expression level was analyzed using comparative 2^−ΔΔCt^ method^52^.

## Electronic supplementary material

Supplementary Information
