# Supplementary material for: Unravelling miRNA regulation in yield of rice (Oryza sativa) based on differential network model
Source: Sci Rep. 2018 May 31;8:8498. doi: 10.1038/s41598-018-26438-w (PMC5981461; doi:10.1038/s41598-018-26438-w)
Supplement: Supplementary file 1 — Supplementary Information [file 41598_2018_26438_MOESM1_ESM.pdf]

Supplemental Information

Unravelling miRNA regulation in yield of rice (*Oryza sativa*) based on differential network model

Jihong Hu[1,2#], Tao Zeng[3#], Qiongmei Xia[4], Qian Qian[2], Congdang Yang[4], Yi Ding[2*], Luonan Chen[3*], Wen Wang[1,5*]

Supplemental Materials and Methods

Plant materials, high-throughput sequencing and bioinformatics analysis

Small RNA and transcriptome sequencing were performed for the variety IR64 in Taoyuan (ultra-high yield) and Jinghong (natural yield) at three different stages (tiller, young panicle and flag leaf) for the yield of rice. Total RNA was extracted from three tissues (tillers, young panicles and flag leaves) of rice IR64 using the Trizol (Invitrogen). For each sample, 5µg of total RNA was used as the input material for constructing the small RNA library. Sequencing libraries were generated using NEBNext Multiplex Small RNALibrary Prep Set for Illumina (NEB, USA) according to the manufacturer's recommendations. Briefly, the small RNAs were ligated with 3' and 5'adapters using T4 RNA ligase. The RNAs were subsequentlytranscribed to single-stranded cDNA using Super-Script II Reverse Transcriptase (RNase H-) (Invitrogen). Then, PCR amplification was performed using LongAmp Taq2XMaster Mix and primers that anneal to adapters. PCR products were purified on an 8 % polyacrylamidegel (100V, 80 min). After quality assessment using DNA High Sensitivity Chips with the Agilent 2100 Bioanalyzer, DNA fragments 140–160 bpinlength were recovered and dissolved in 8 µl of elutionbuffer for sequencing. Then, the libraries were sequenced using the Illumina HiSeq2500 platform with single-end (SE) 50 nt.

After Illumina sequencing, any low quality reads, adaptors, contaminating sequences and sequences shorter than 18 nt were discarded. Only the remaining high-quality sequences between 18 and 30 nt were further analyzed. All unique sequences were aligned to the rice genome (Nipponbare-Reference-IRGSP-1.0) and annotated based on MSU-v7.0 (http://rice.plantbiology.msu.edu/) using SOAP for mapping (Li et al., 2008). Reads that mapped to rice rRNA, tRNA, scRNA, snRNA, or snoRNA were removed based on the National Center for Biotechnology Information (NCBI) (http://www.ncbi.nlm.nih.gov/) and Rfam RNA family databases (Gardner et al., 2009). Known miRNAs were identified using a BLAST search against the miRNA database miRBase release 21 (http://mirbase.org/)

(Kozomara and Griffiths-Jones, 2014). Reads that did not annotate to any category were used to predict novel miRNAs using the miRNA prediction program MIREAP (http://sourceforge.net/projects/mireap/) as described (Yan et al., 2015). Secondary structures of potential miRNA precursors were constructed using the MFOLD3.2 web server (http://mfold.rna.albany.edu/) (Zuker, 2003).To identify miRNAs with differential expression between Taoyuan and Jinghong in the three tissues, miRNA read counts were normalized to transcripts per million (TPM) using the following formula: normalized expression = (miRNA count / total count of clean reads) $\times 10^6$ (Hu et al., 2016).

For RNA sequencing (RNA-seq) library construction, 3 μg of total RNA of each sample was used for library preparation using TruSeq Stranded Total RNA Sample Preparation kit (Illumina, San Diego, USA). RNA was fragmented into small pieces and then first-strand cDNA was synthesized with Super Script Ⅱ reverse transcription (Invitrogen). After purifying, the second strand cDNA library was synthesized, following several rounds of PCR amplification. The clean reads of RNA-seq were mapped to the rice reference genome (Nipponbare-Reference-IRGSP-1.0) using Tophat 2 (version 2.0.13) (Trapnell et al., 2010). Transcript reconstruction was conducted by Cufflinks software (version 2.2.1) (Trapnell et al., 2012). And DESeq2 was used to make read counts and to identify differentially expressed genes (DEGs). A False Discovery Rate (FDR) was determined with threshold of 0.05 and |log2(fold change)| ≥1) to recognize the significance of the gene expression difference. All the small and RNA sequencing data were deposited in the NCBI Short Read Archive (SRA) (http://www.ncbi.nlm.nih.gov/sra) under the accession number: SRP134071 and SRP144409.

Differential edge-like transformation (DET)

The differential co-expression analysis is to see if or not the expression correlation of a gene-pair (e.g. two genes or molecules) changes between control and case samples (Zeng et al., 2014; Yu et al., 2015). Thus, the Pearson correlation coefficient (PCC) between genes *I* and *j* in control or case samples can be calculated.

On one hand, to evaluate the correlation in one sample, the pseudo-correlation or correlation-like expressionof such a gene-pair in a sample *k* can be defined as:

Pseudo-correlation in control condition: $\frac{x_{\mathrm{ik}}-\mu_{\mathrm{xi}}}{\sigma_{\mathrm{xi}}} \cdot \frac{x_{\mathrm{jk}}-\mu_{\mathrm{xj}}}{\sigma_{\mathrm{xj}}} = \mathrm{C}(x_{\mathrm{ik}})\mathrm{C}(x_{\mathrm{jk}})$ (1)

Pseudo-correlation in case condition: $\frac{y_{\mathrm{ik}}-\mu_{\mathrm{yi}}}{\sigma_{\mathrm{yi}}} \cdot \frac{y_{\mathrm{jk}}-\mu_{\mathrm{yj}}}{\sigma_{\mathrm{yj}}} = \mathrm{C}(y_{\mathrm{ik}})\mathrm{C}(y_{\mathrm{jk}})$ (2)

Where, C (.) represents the Z-transform of one sample, i.e., sample k, by the mean and variance in such sample's group (e.g. a control sample and control group). Comparing (1) and (2), clearly for one gene-pair, the mean of its pseudo-correlation in all control (or case) samples just equals to the Pearson correlation coefficienton control (or case) group (Zeng et al., 2014; Zhang et al., 2015).

On the other hand, to evaluate the correlation change of one sample compared to multiple controls, the delta expression of a gene-pair can be computed as:

Pseudo-correlation in control condition: $\frac{x_{\mathrm{i}}-\mu_{\mathrm{xi}}}{\sigma_{\mathrm{xi}}} \cdot \frac{x_{\mathrm{j}}-\mu_{\mathrm{xj}}}{\sigma_{\mathrm{xj}}} = \mathrm{C}(x_{\mathrm{i}})\mathrm{C}(x_{\mathrm{j}})$

Delta-correlationin case condition: $\frac{y_{\mathrm{i}}-\mu_{\mathrm{xi}}}{\sigma_{\mathrm{xi}}} \cdot \frac{y_{\mathrm{j}}-\mu_{\mathrm{xj}}}{\sigma_{\mathrm{xj}}} = \mathrm{C}'(\mathrm{y_i})\mathrm{C}'(y_{\mathrm{j}})$ (3)

Where, C (.) represents the Z-transform of one sample by the mean and variance in such sample's group (e.g. a control sample againstthe control group); and C' (.) represents the Z-transform of one sample by the mean and variance in another group (e.g. a case sample against the control group, or vice versa). According to the SSN theory (Liu et al., 2016), such delta-correlation would reflect the significance of correlation (network) change when one case sample mixed in a group of control samples (called reference samples in Liu et al., 2016).

Obviously, the first method (eqn. (2)) solves the weight representation of a gene-pair (e.g. an edge in network) in one sample but doesnot consider the unbalance of sample numbers between control and case samples. Meanwhile, the second method(eqn.(3)) gives a direct alterationrepresentation for a case sample, however, such measurement for control and case samples would have different correlation meaning, i.e. the mean of delta correlation of a gene-pair in control samples would be the Pearson correlation coefficienton all control samples, but it is not on all case samples.

Thus, to provide a more comprehensive approach to evaluate the network, i.e. the edges / gene-pairs, in one sample, we here introduce a new differential edge-like transformation (DET) such as:

Edge-like correlation in control condition:

$$\frac{x_{\mathrm{ik}}-\mu_{\mathrm{xyi}}}{\sigma_{\mathrm{xyi}}}\cdot\frac{x_{\mathrm{jk}}-\mu_{\mathrm{xyj}}}{\sigma_{\mathrm{xyj}}} = \lambda_1\cdot \mathrm{C}(x_{\mathrm{ik}})\mathrm{C}(x_{\mathrm{jk}}) + \lambda_2\cdot \mathrm{C}(x_{\mathrm{ik}})\mathrm{C}'(x_{\mathrm{jk}}) + \lambda_3\cdot \mathrm{C}'(x_{\mathrm{ik}})\mathrm{C}(x_{\mathrm{jk}}) + \lambda_4 \cdot \mathrm{C}'(x_{\mathrm{ik}})\mathrm{C}'(x_{\mathrm{jk}})$$

Edge-like correlation in case condition:

$$\frac{y_{\mathrm{ik}}-\mu_{\mathrm{xyi}}}{\sigma_{\mathrm{xyi}}}\cdot\frac{y_{\mathrm{jk}}-\mu_{\mathrm{xyj}}}{\sigma_{\mathrm{xyj}}} = \lambda_1\cdot \mathrm{C}'(y_{\mathrm{ik}})\mathrm{C}'(y_{\mathrm{jk}}) + \lambda_2\cdot \mathrm{C}'(y_{\mathrm{ik}})\mathrm{C}(y_{\mathrm{jk}}) + \lambda_3\cdot \mathrm{C}(y_{\mathrm{ik}})\mathrm{C}'(y_{\mathrm{jk}}) + \lambda_4 \cdot \mathrm{C}(y_{\mathrm{ik}})\mathrm{C}(y_{\mathrm{jk}})$$

Where, $\lambda_1=\frac{\sigma_{\mathrm{xi}}\sigma_{\mathrm{xj}}}{4\sigma_{\mathrm{xyi}}\sigma_{\mathrm{xyj}}},\lambda_2=\frac{\sigma_{\mathrm{xi}}\sigma_{\mathrm{yj}}}{4\sigma_{\mathrm{xyi}}\sigma_{\mathrm{xyj}}},\lambda_3=\frac{\sigma_{\mathrm{yi}}\sigma_{\mathrm{xj}}}{4\sigma_{\mathrm{xyi}}\sigma_{\mathrm{xyj}}},\lambda_4=\frac{\sigma_{\mathrm{yi}}\sigma_{\mathrm{yj}}}{4\sigma_{\mathrm{xyi}}\sigma_{\mathrm{xyj}}}$, and there are similar numbers of control and case samples.

DET transforms the expression of genes to the edge-like correlation of gene-pairs in one sample, and the mean of edge-like correlation of a gene-pair in all control and case samples is just the Pearson correlation coefficienton all samples, so that this measurement has equivalent numerical meaning for any control or case sample. In addition, according to the following theory 1, such a transformation also has other two compatiblefeatures with previous methods (e.g. above pseudo-correlation and delta-correlation):

(i) When the sample numbers are extremely unbalance between control and case, the edge-like correlation will approach to delta correlation;

(ii) When the genes have the equivalent expression variances between control and casegroups, the edge-like correlation will be a linear combination among psesudo-correlation and delta-correlation; otherwise, the edge-like correlation will have weighted variants for control and case samples, respectively.

Totally, DET can provide a more general approach to evaluate the network, i.e. the edges / gene-pairs, in one sample, under more complex practical conditions, such as: the sample numbers are extremely unbalance, or the genes have differential variances / co-variances.

Theoretical Result: Assume that gene *i* and *j* have one-sample's representative expressions $\mathrm{x_i}$ and $\mathrm{x_j}$ in control with m_{x} samples, and $\mathrm{y_i}$ and $\mathrm{y_j}$ in case with m_{y} samples, the edge-like correlation of a gene-pair between *i* and *j* in one sample can be obtained as a transformation:

$$\frac{x_i-\mu_{xyi}}{\sigma_{xyi}}\cdot\frac{x_j-\mu_{xyj}}{\sigma_{xyj}}$$

$$\frac{y_i-\mu_{xyi}}{\sigma_{xyi}}\cdot\frac{y_j-\mu_{xyj}}{\sigma_{xyj}}$$

Then,

(i) when $\frac{m_x}{m_y}\to\infty$,

$$\frac{x_i-\mu_{xyi}}{\sigma_{xyi}}\cdot\frac{x_j-\mu_{xyj}}{\sigma_{xyj}}\to\frac{\sigma_{xi}\sigma_{xj}}{\sigma_{xyi}\sigma_{xyj}}\cdot\left(\frac{x_i-\mu_{xi}}{\sigma_{xi}}\cdot\frac{x_j-\mu_{xj}}{\sigma_{xj}}\right)$$

$$\frac{y_i-\mu_{xyi}}{\sigma_{xyi}}\cdot\frac{y_j-\mu_{xyj}}{\sigma_{xyj}}\to\frac{\sigma_{xi}\sigma_{xj}}{\sigma_{xyi}\sigma_{xyj}}\cdot\left(\frac{x_i-\mu_{xi}}{\sigma_{xi}}\cdot\frac{x_j-\mu_{xj}}{\sigma_{xj}}\right);$$

(ii) When $\frac{m_x}{m_y}\to 1$ and $\sigma_{xi}\to\sigma_{yi}$ and $\sigma_{xj}\to\sigma_{yj}$,

$$\frac{x_i-\mu_{xyi}}{\sigma_{xyi}}\cdot\frac{x_j-\mu_{xyj}}{\sigma_{xyj}}\to\lambda\cdot[C(x_i)C(x_j)+C(x_i)C'(x_j)+C'(x_i)C(x_j)+C'(x_i)C'(x_j)]$$

$$\frac{y_i-\mu_{xyi}}{\sigma_{xyi}}\cdot\frac{y_j-\mu_{xyj}}{\sigma_{xyj}}\to\lambda\cdot[C(y_i)C(y_j)+C(y_i)C'(y_j)+C'(y_i)C(y_j)+C'(y_i)C'(y_j)].$$

Where, $\lambda=\frac{\sigma_{xi}\sigma_{xj}}{4\sigma_{xyi}\sigma_{xyj}}$; the expression average and variance for gene *i* (or gene *j*) on control samples are μ_{xi} and σ_{xi} (or μ_{xj} and σ_{xj}); and conveniently, the sample in case has these similar variables and annotations; and μ_{xyi} and σ_{xyi}(or μ_{xyj} and σ_{xyj}) are the mean and variance of gene *i* (or gene *j*) in all control and case samples.

Proof:

According to the relation between sample means in control, case and all, there are:

$$\mu_{xyi}=\left(\frac{m_x}{m}\right)\mu_{xi}+\left(\frac{m_y}{m}\right)\mu_{yi}$$

$$\mu_{xyj}=\left(\frac{m_x}{m}\right)\mu_{xj}+\left(\frac{m_y}{m}\right)\mu_{yj}$$

where $m=m_x+m_y$, so,

$$\frac{x_i-\mu_{xyi}}{\sigma_{xyi}}\cdot\frac{x_j-\mu_{xyj}}{\sigma_{xyj}}=\frac{\sigma_{xi}\sigma_{xj}}{\sigma_{xyi}\sigma_{xyj}}\cdot\left(\frac{x_i-\mu_{xyi}}{\sigma_{xi}}\cdot\frac{x_j-\mu_{xyj}}{\sigma_{xj}}\right)$$

$$=\frac{\sigma_{xi}\sigma_{xj}}{\sigma_{xyi}\sigma_{xyj}}\cdot\left(\frac{x_i-\left(\frac{m_x}{m}\right)\mu_{xi}-\left(\frac{m_y}{m}\right)\mu_{yi}}{\sigma_{xi}}\cdot\frac{x_j-\left(\frac{m_x}{m}\right)\mu_{xj}-\left(\frac{m_y}{m}\right)\mu_{yj}}{\sigma_{xj}}\right)$$

$$\frac{y_i-\mu_{xyi}}{\sigma_{xyi}}\cdot\frac{y_j-\mu_{xyj}}{\sigma_{xyj}}=\frac{\sigma_{xi}\sigma_{xj}}{\sigma_{xyi}\sigma_{xyj}}\cdot\left(\frac{y_i-\mu_{xyi}}{\sigma_{xi}}\cdot\frac{y_j-\mu_{xyj}}{\sigma_{xj}}\right)$$

$$=\frac{\sigma_{xi}\sigma_{xj}}{\sigma_{xyi}\sigma_{xyj}}\cdot\left(\frac{y_i-\left(\frac{m_x}{m}\right)\mu_{xi}-\left(\frac{m_y}{m}\right)\mu_{yi}}{\sigma_{xi}}\cdot\frac{y_j-\left(\frac{m_x}{m}\right)\mu_{xj}-\left(\frac{m_y}{m}\right)\mu_{yj}}{\sigma_{xj}}\right).$$

Then following results hold,

(i) When $\frac{m_x}{m_y} \to \infty$,

there will be $\frac{m_x}{m} \to 1$ and $\frac{m_y}{m} \to 0$, and

$$\frac{x_i - \mu_{xyi}}{\sigma_{xyi}} \cdot \frac{x_j - \mu_{xyj}}{\sigma_{xyj}} \to \frac{\sigma_{xi}\sigma_{xj}}{\sigma_{xyi}\sigma_{xyj}} \cdot \left(\frac{x_i - \mu_{xi}}{\sigma_{xi}} \cdot \frac{x_j - \mu_{xj}}{\sigma_{xj}}\right)$$

$\frac{y_i-\mu_{xyi}}{\sigma_{xyi}} \cdot \frac{y_j-\mu_{xyj}}{\sigma_{xyj}} \to \frac{\sigma_{xi}\sigma_{xj}}{\sigma_{xyi}\sigma_{xyj}} \cdot \left(\frac{y_i-\mu_{xi}}{\sigma_{xi}} \cdot \frac{y_j-\mu_{xj}}{\sigma_{xj}}\right)$.

Under this condition, the edge-like correlation will approach to delta-correlation (with a scale).

Similarly, when $\frac{m_x}{m_y} \to 0$, it can be dealt as $\frac{m_y}{m_x} \to \infty$.

(ii) When$\frac{m_x}{m_y} \to 1$,

there will be $\frac{m_x}{m} \to \frac{1}{2}$ and $\frac{m_y}{m} \to \frac{1}{2}$, and

$$\frac{x_i - \mu_{xyi}}{\sigma_{xyi}} \cdot \frac{x_j - \mu_{xyj}}{\sigma_{xyj}} = \frac{\sigma_{xi}\sigma_{xj}}{\sigma_{xyi}\sigma_{xyj}} \cdot \left(\frac{2x_i - \mu_{xi} - \mu_{yi}}{2\sigma_{xi}} \cdot \frac{2x_j - \mu_{xj} - \mu_{yj}}{2\sigma_{xj}}\right)$$

$$= \frac{\sigma_{xi}\sigma_{xj}}{\sigma_{xyi}\sigma_{xyj}} \cdot \left(\frac{(x_i - \mu_{xi}) + (x_i - \mu_{yi})}{2\sigma_{xi}} \cdot \frac{(x_j - \mu_{xj}) + (x_j - \mu_{yj})}{2\sigma_{xj}}\right)$$

$$= \frac{\sigma_{xi}\sigma_{xj}}{4\sigma_{xyi}\sigma_{xyj}} \cdot \left(\left(\frac{(x_i - \mu_{xi})}{\sigma_{xi}} + \frac{(x_i - \mu_{yi})}{\sigma_{xi}}\right) \cdot \left(\frac{(x_j - \mu_{xj})}{\sigma_{xj}} + \frac{(x_j - \mu_{yj})}{\sigma_{xj}}\right)\right)$$

$$= \frac{\sigma_{xi}\sigma_{xj}}{4\sigma_{xyi}\sigma_{xyj}} \cdot \left(\frac{(x_i - \mu_{xi})}{\sigma_{xi}} \cdot \frac{(x_j - \mu_{xj})}{\sigma_{xj}} + \frac{(x_i - \mu_{xi})}{\sigma_{xi}} \cdot \frac{(x_j - \mu_{yj})}{\sigma_{xj}} + \frac{(x_i - \mu_{yi})}{\sigma_{xi}} \cdot \frac{(x_j - \mu_{xj})}{\sigma_{xj}} + \frac{(x_i - \mu_{yi})}{\sigma_{xi}} \cdot \frac{(x_j - \mu_{yj})}{\sigma_{xj}}\right)$$

$$= \frac{\sigma_{xi}\sigma_{xj}}{4\sigma_{xyi}\sigma_{xyj}} \cdot \frac{(x_i - \mu_{xi})}{\sigma_{xi}} \cdot \frac{(x_j - \mu_{xj})}{\sigma_{xj}} + \frac{\sigma_{xi}\sigma_{yj}}{4\sigma_{xyi}\sigma_{xyj}} \cdot \frac{(x_i - \mu_{xi})}{\sigma_{xi}} \cdot \frac{(x_j - \mu_{yj})}{\sigma_{yj}} + \frac{\sigma_{yi}\sigma_{xj}}{4\sigma_{xyi}\sigma_{xyj}} \cdot \frac{(x_i - \mu_{yi})}{\sigma_{yi}} \cdot \frac{(x_j - \mu_{xj})}{\sigma_{xj}} + \frac{\sigma_{yi}\sigma_{yj}}{4\sigma_{xyi}\sigma_{xyj}} \cdot \frac{(x_i - \mu_{yi})}{\sigma_{yi}} \cdot \frac{(x_j - \mu_{yj})}{\sigma_{yj}}$$

$=\lambda_1 \cdot C(x_i)C(x_j) + \lambda_2 \cdot C(x_i)C'(x_j) + \lambda_3 \cdot C'(x_i)C(x_j) + \lambda_4 \cdot C'(x_i)C'(x_j)$.

Where, $\lambda_1 = \frac{\sigma_{xi}\sigma_{xj}}{4\sigma_{xyi}\sigma_{xyj}}, \lambda_2 = \frac{\sigma_{xi}\sigma_{yj}}{4\sigma_{xyi}\sigma_{xyj}}, \lambda_3 = \frac{\sigma_{yi}\sigma_{xj}}{4\sigma_{xyi}\sigma_{xyj}}, \lambda_4 = \frac{\sigma_{yi}\sigma_{yj}}{4\sigma_{xyi}\sigma_{xyj}}$

Similarly, there is,

$$\frac{y_i - \mu_{xyi}}{\sigma_{xyi}} \cdot \frac{y_j - \mu_{xyj}}{\sigma_{xyj}} = \frac{\sigma_{yi}\sigma_{yj}}{\sigma_{xyi}\sigma_{xyj}} \cdot \left(\frac{2y_i - \mu_{xi} - \mu_{yi}}{2\sigma_{yi}} \cdot \frac{2y_j - \mu_{xj} - \mu_{yj}}{2\sigma_{yj}}\right)$$

$= \lambda_1 \cdot C'(y_i)C'(y_j) + \lambda_2 \cdot C'(y_i)C(y_j) + \lambda_3 \cdot C(y_i)C'(y_j) + \lambda_4 \cdot C(y_i)C(y_j)$.

In addition, if $\sigma_{xi} \to \sigma_{yi}$ and $\sigma_{xj} \to \sigma_{yj}$, the above measurements can be further reduced to

$$\frac{x_i - \mu_{xyi}}{\sigma_{xyi}} \cdot \frac{x_j - \mu_{xyj}}{\sigma_{xyj}} \to \lambda \cdot [C(x_i)C(x_j) + C(x_i)C'(x_j) + C'(x_i)C(x_j) + C'(x_i)C'(x_j)]$$

$$\frac{y_i - \mu_{xyi}}{\sigma_{xyi}} \cdot \frac{y_j - \mu_{xyj}}{\sigma_{xyj}} \to \lambda \cdot [C(y_i)C(y_j) + C(y_i)C'(y_j) + C'(y_i)C(y_j) + C'(y_i)C'(y_j)].$$

Where, $\lambda = \frac{\sigma_{xi}\sigma_{xj}}{4\sigma_{xyi}\sigma_{xyj}}$.

Thus, under this condition, the edge-like correlation will be a linear combination among pseudo-correlation and delta-correlation and their combinations, similarly for control or case samples.

Gene regulatory networks with DET

Based on the theory of differential edge-like transformation (DET), a new differential network model by combining the miRNA-mRNA regulatory network and its edge-like correlationsis proposed in this study (Fig. 1).

In one condition or in one tissue, there is usually only one sample, the conventional correlation-based approaches (e.g. WGCNA) will not be applicable. By contrast, we applied DET to capture the significant gene expression and correlation changes simultaneously in a dynamical and network manner. Assume that the (miRNA & mRNA) expression data $X=[x_1,x_2,...x_N]$ with $x_i=[x_{i1},x_{i2},...x_{iM}]$ are an expression profile of a miRNA or a mRNA across a group of tissues,

(i) the tissue-specific expression of molecule *i* on one tissue *k* can be obtained by:

$$\overline{x}_{i,k} = \frac{x_{ik} - \text{mean}(x_i)}{\text{std}(x_i)}$$

(ii) the tissue-specific edge-like correlation of molecules *i* and *j* on one tissue *k* can be obtained by (given molecule *i* is a miRNA and molecule *j* is a target mRNA):

$$\overline{\rho}_{ij,k} = \frac{x_{ik} - \text{mean}(x_i)}{\text{std}(x_i)} \cdot \frac{x_{jk} - \text{mean}(x_j)}{\text{std}(x_j)}$$

Simply, the differential molecule or tissue-specific molecule on one tissue*k* can be selected by Mann–Whitney U test between $[\overline{x}_{i,k}]$and $[\overline{x}_{i,1}, \ldots, \overline{x}_{i,k-1}, \overline{x}_{i,k+1}, \ldots]$. And the differential molecule-pairs or sample-specific molecule association on one sample *k* can be selected by

Mann–Whitney U test between $[\overline{\rho}_{ij,k}]$and $[\overline{\rho}_{ij,1}, \dots, \overline{\rho}_{ij,k-1}, \overline{\rho}_{ij,k+1}, \dots]$. For any miRNA, its average PCC (i.e. the edge-like correlation) with other relevant miRNAs or mRNAs in control or case is defined as $AP_{control}$ and AP_{case}, then a factor as PCC-induced key-associated score for this miRNA is computed as $|AP_{case} - AP_{control}|$.

Supplemental References

Gardner, P.P. *et al.* Rfam: updates to the RNA families database. *Nucleic Acids Res.* **37**, 136-140 (2009).

Hu, J.H., Jin, J., Qian, Q., Huang, K.K. & Ding, Y. Small RNA and degradome profiling reveals miRNA regulation in the seed germination of ancient eudicot *Nelumbo nucifera*. *BMC Genomics* **17**, 684 (2016).

Kozomara, A., & Griffiths, J.S. miRBase: integrating microRNA annotation and deep-sequencing data. *Nucleic Acids Res.* **42**, D68-D73(2014).

Li, R.Q., Li, Y.R., Kristiansen, K, & Wang, J. SOAP: short oligonucleotide alignment program. *Bioinformatics* **24**, 713-714(2008).

Liu, L. *et al.* An integrative bioinformatics framework for genome-scale multiple level network reconstruction of rice. *J. Inegr. Bioinformatics* **10**, 10.2390 (2013).

Liu, X., Wang, Y., Ji, H., Aihara, K., & Chen, L. Personalized characterization of diseases using sample-specific networks. *Nucleic Acids Res.* **44**(22), e164 (2016).

Trapnell, C. *et al.* Differential gene and transcript expression analysis of RNA-seq experiments with TopHat and Cufflinks. *Nature Protoc.* **7**, 562–578 (2012).

Trapnell, C. *et al.* Transcript assembly and quantification by RNA-Seq reveals unannotated transcripts and isoform switching during cell differentiation. *Nat. Biotechnol.* **28**, 511–515 (2010).

Yan, J.J., Zhang, H.Y., Zheng, Y.Z. & Ding, Y. Comparative expression progiling of miRNAs between the cytoplasmic male sterile line MeixiangA and its maintianer line MeixiangB during rice anther development. *Planta* **241**, 109-123 (2015).

Yu, X., Zeng, T., Wang, X., Li, G. & Chen L. Unravelling personalized dysfunctional gene network of complex diseases based on differential network model. *J. Transl. Med.***13**, 189 (2015).

Zeng, T. *et al.* Edge biomarkers for classification and prediction of phenotypes. *Sci China Life Sci.* **57**(11), 1103-14 (2014).

Zhang, W., Zeng, T., Liu, X. & Chen, L. Diagnosing phenotypes of single-sample individuals by edge biomarkers. *J. Mol. Cell Biol.* **7**(3), 231-241 (2015).

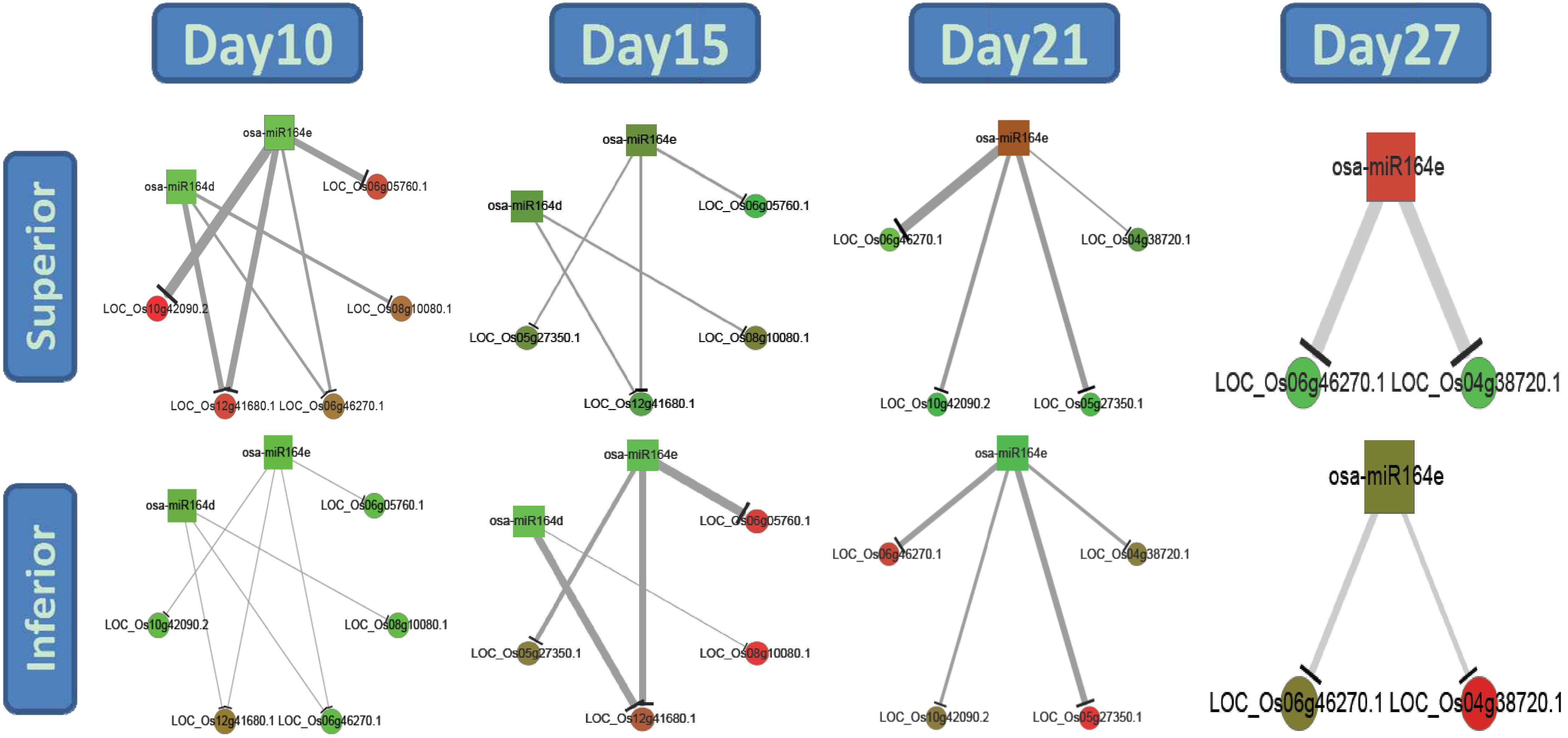
Day10
Day15
Day21
Day27
Superior
Inferior
osa-miR164e
osa-miR164d
LOC_Os06g05760.1
LOC_Os10g42090.2
LOC_Os08g10080.1
LOC_Os12g41680.1
LOC_Os06g46270.1
LOC_Os05g27350.1
LOC_Os04g38720.1

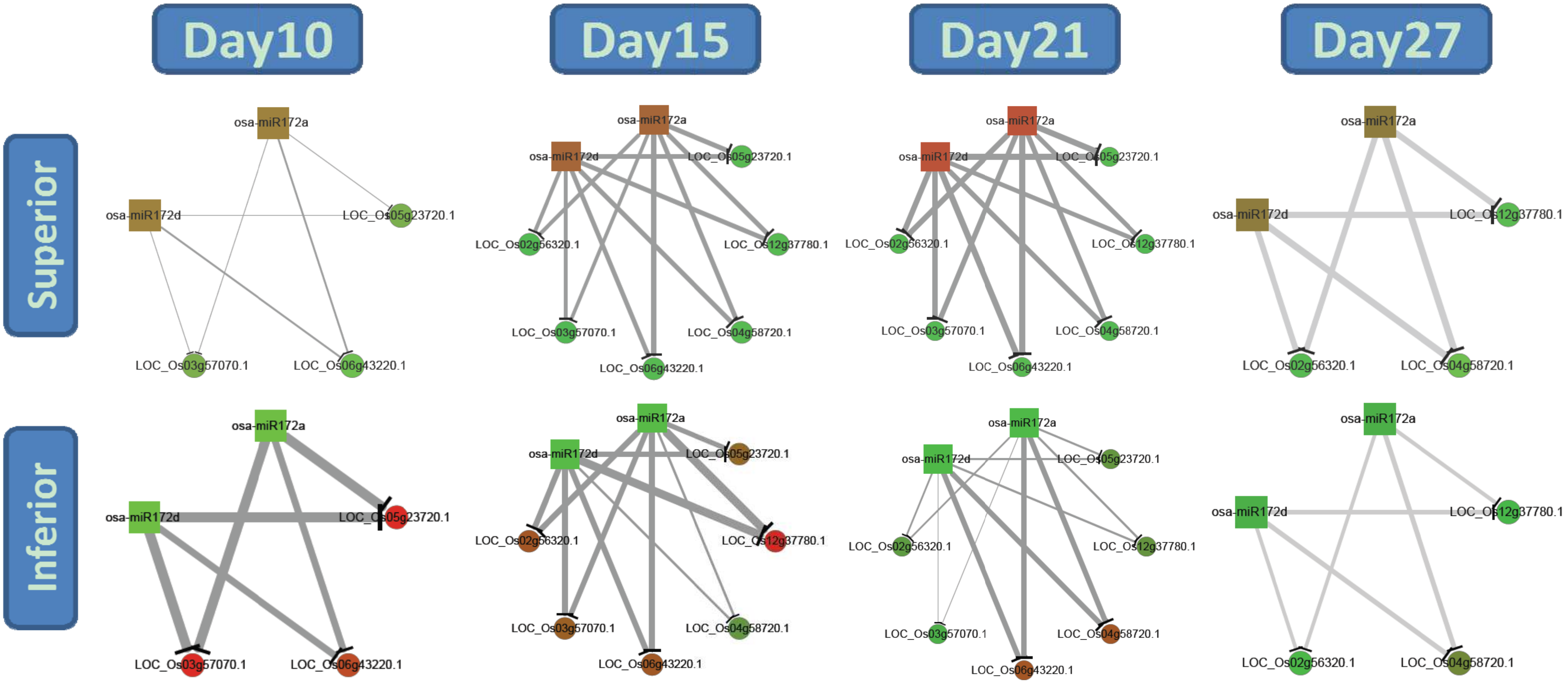

Day10
Day15
Day21
Day27
Superior
Inferior
osa-miR172a
osa-miR172d
LOC_Os05g23720.1
LOC_Os02g56320.1
LOC_Os12g37780.1
LOC_Os03g57070.1
LOC_Os04g58720.1
LOC_Os06g43220.1

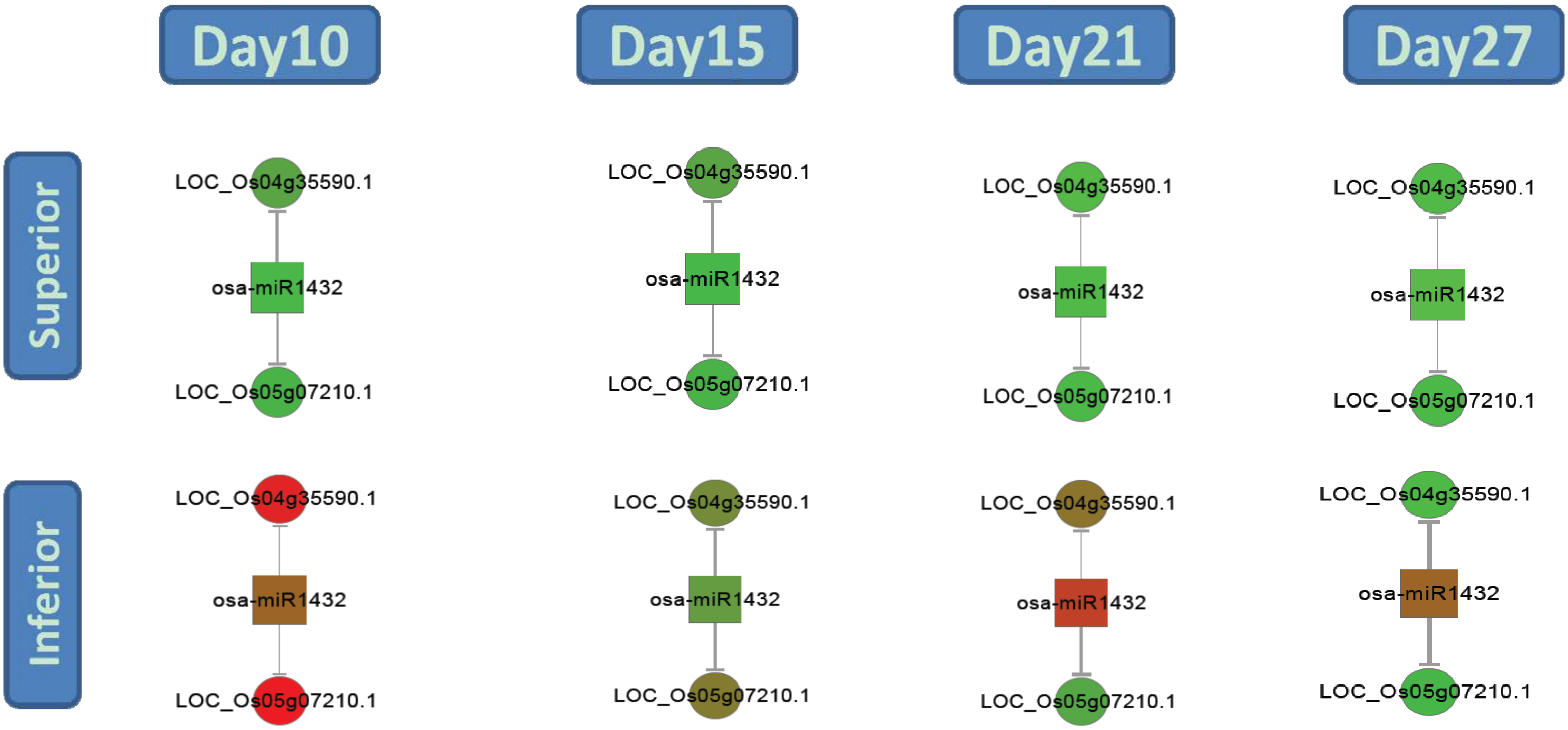

Supplementary Fig. S1 Subnetwork of differentially expressed miRNAs in DAF10, DAF15, DAF21, DAF27 of 'xingfeng 2' rice. The thickness of lines represents the correlation extent of miRNAs and their targets and the thicker, the more negative correlation between them. Rectangles: miRNAs, circles: genes, triangles: TF. The color of them indicates differential expression (red, up-regulation; green, down-regulation).

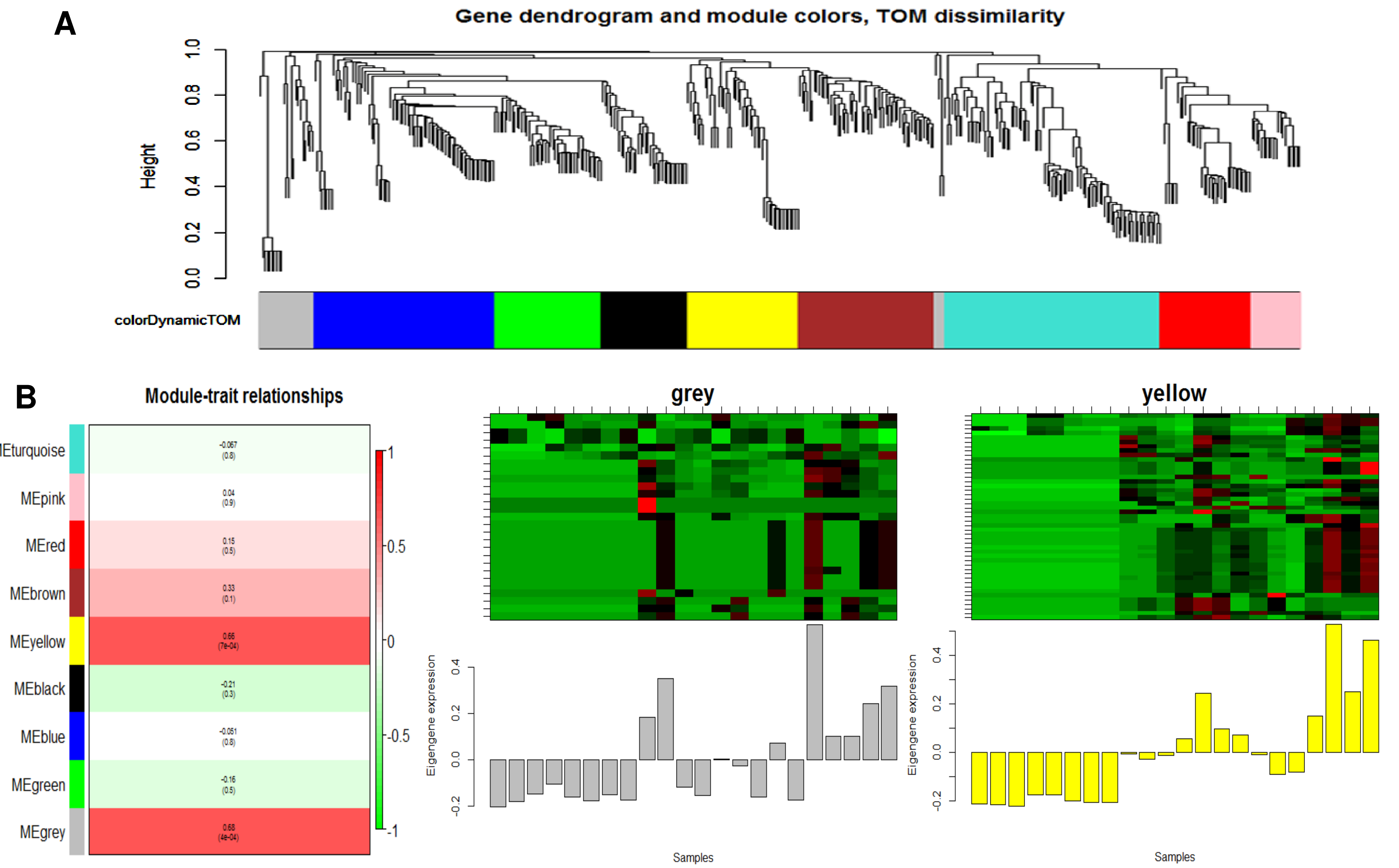
A
Gene dendrogram and module colors, TOM dissimilarity
Height
0.0
0.2
0.4
0.6
0.8
1.0
colorDynamicTOM
B
Module-trait relationships
MEturquoise
MEpink
MEred
MEbrown
MEyellow
MEblack
MEblue
MEgreen
MEgrey
-0.067 (0.8)
0.04 (0.9)
0.15 (0.5)
0.33 (0.1)
0.66 (7e-04)
-0.21 (0.3)
-0.051 (0.8)
-0.16 (0.5)
0.68 (4e-04)
1
0.5
0
-0.5
-1
grey
yellow
Eigengene expression
-0.2
0.0
0.2
0.4
Samples

C

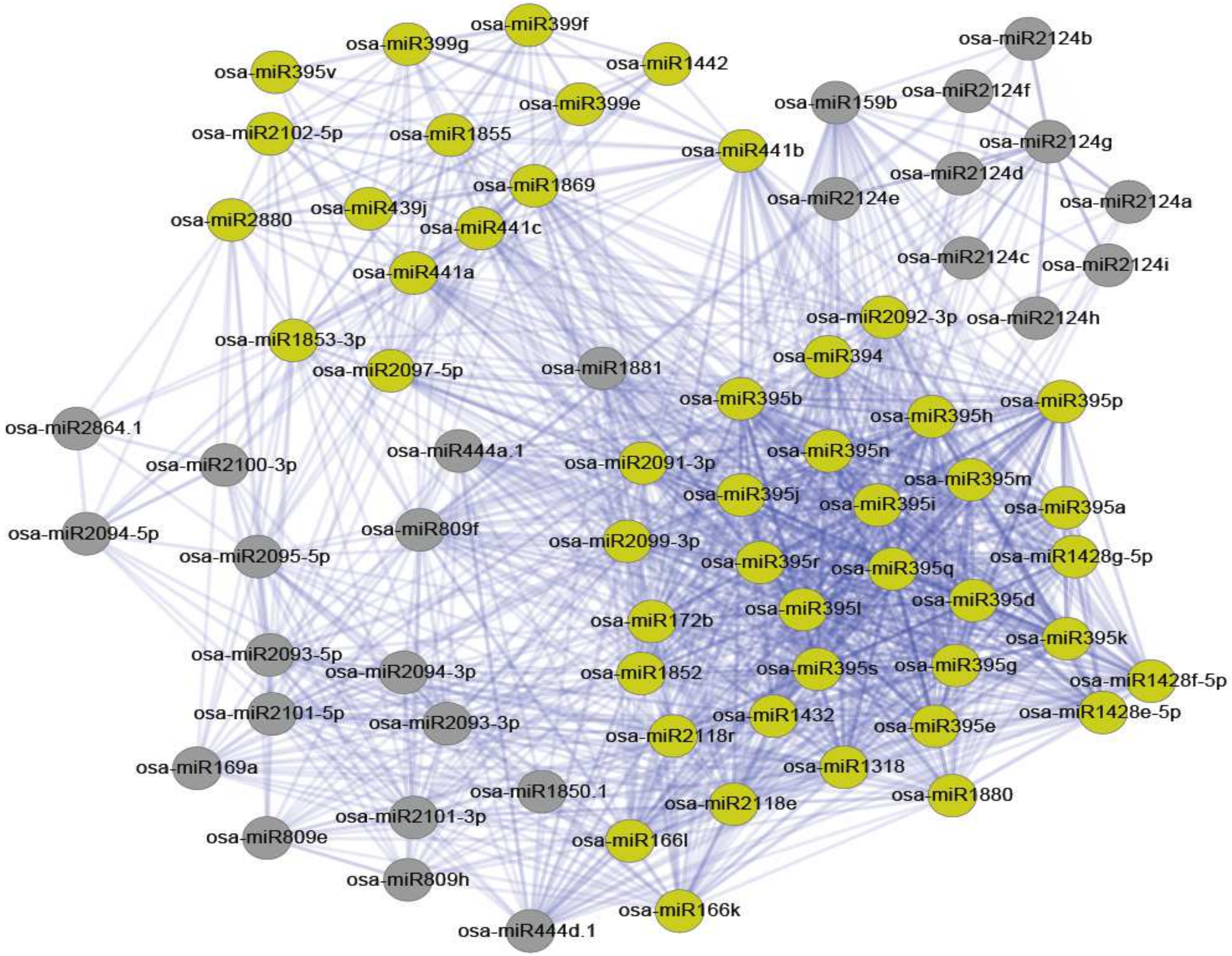

Supplementary Fig. S2 Weighted gene co-expression network analysis (WGCNA) of differentially expressed miRNAs (DEmiRNAs) in superior and inferior spikelets of Xinfeng2 rice. (A) Hierarchical cluster tree showing nine modules of co-expressed miRNAs. (B) Module-grain filling correlations and corresponding *P*-values (left). Heatmaps of the miRNAs expression in "grey" and "yellow" with expression patterns of selected candidate miRNAs (right). (C) Networks of "grey" and "yellow" module. The miRNAs co-expression relationships are colored in "grey" and "yellow", respectively.

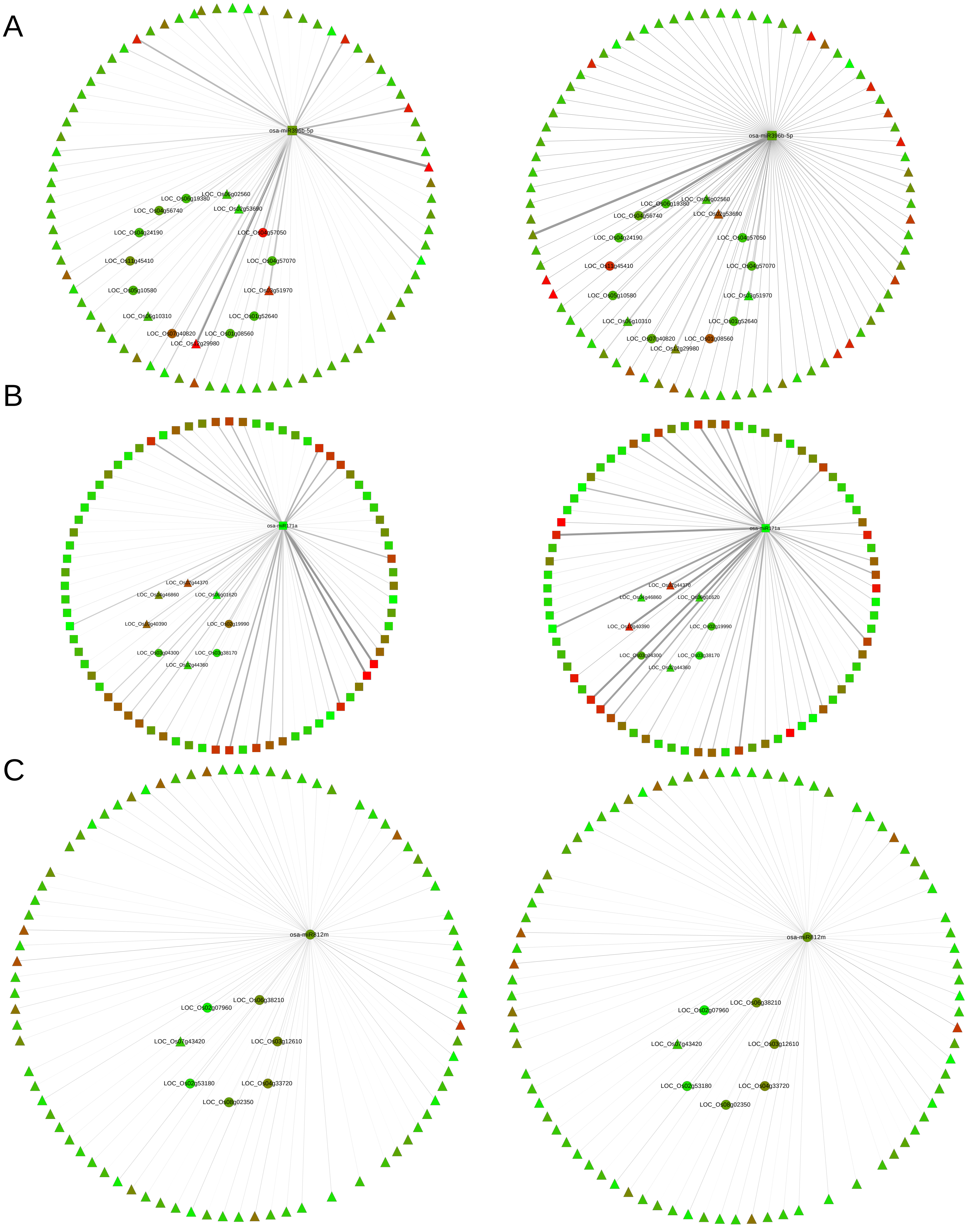

Supplementary Fig. S3 Subnetwork of three miRNAs in tillers, young panicles and flag leaves of Taoyuan (L) and Jinghong (R) IR64 rice. (A) osa-miR396b-5p**, (B)** osa-miR171a**, (C)** osa-miR812m**.** The thickness of lines represents the correlation extent of miRNAs and their targets and the thicker, the more negative correlation between them. Rectangles: miRNAs, circles: genes, triangles: TF. red color, up-regulation and green, down-regulation.

A

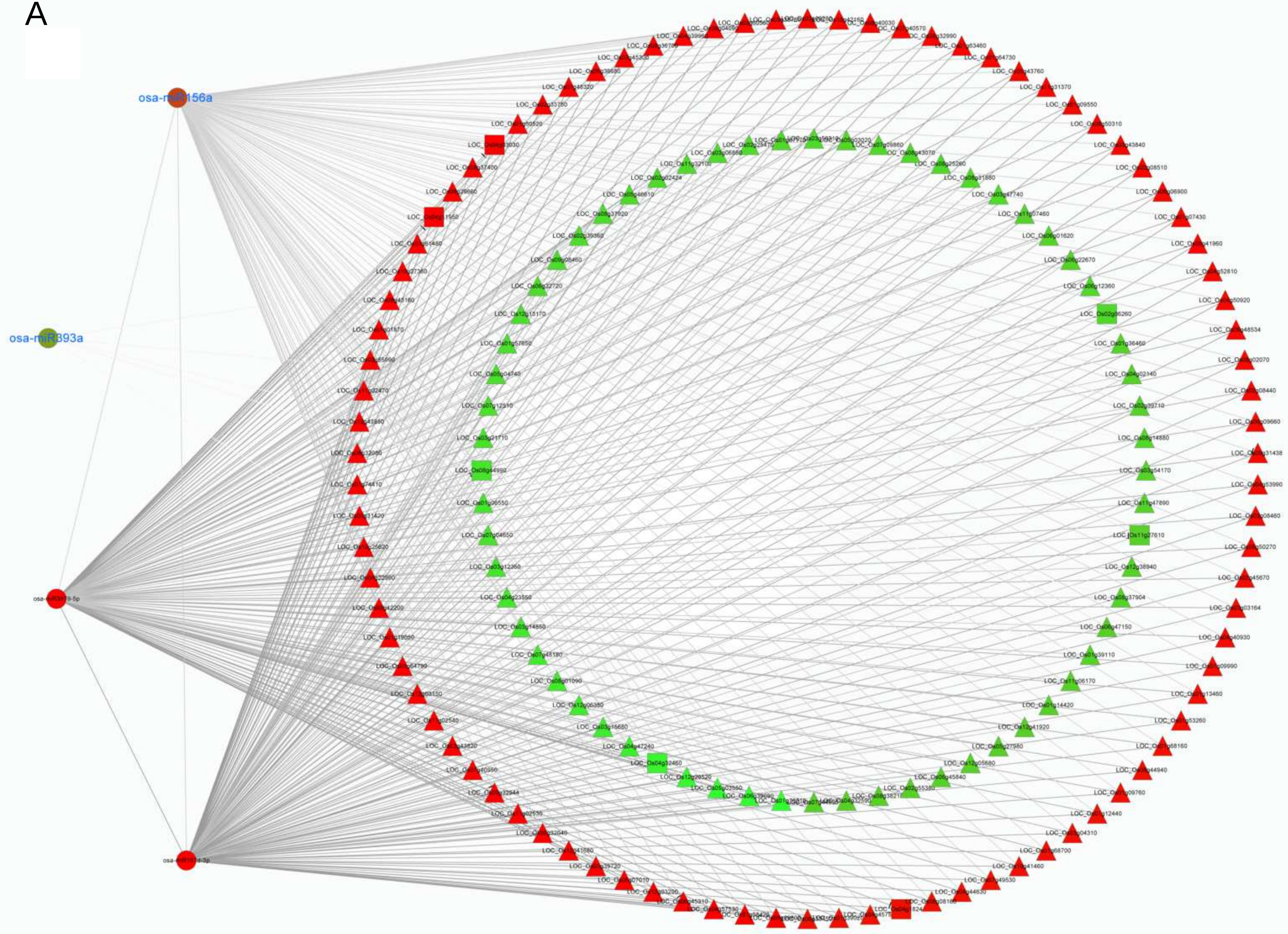

B

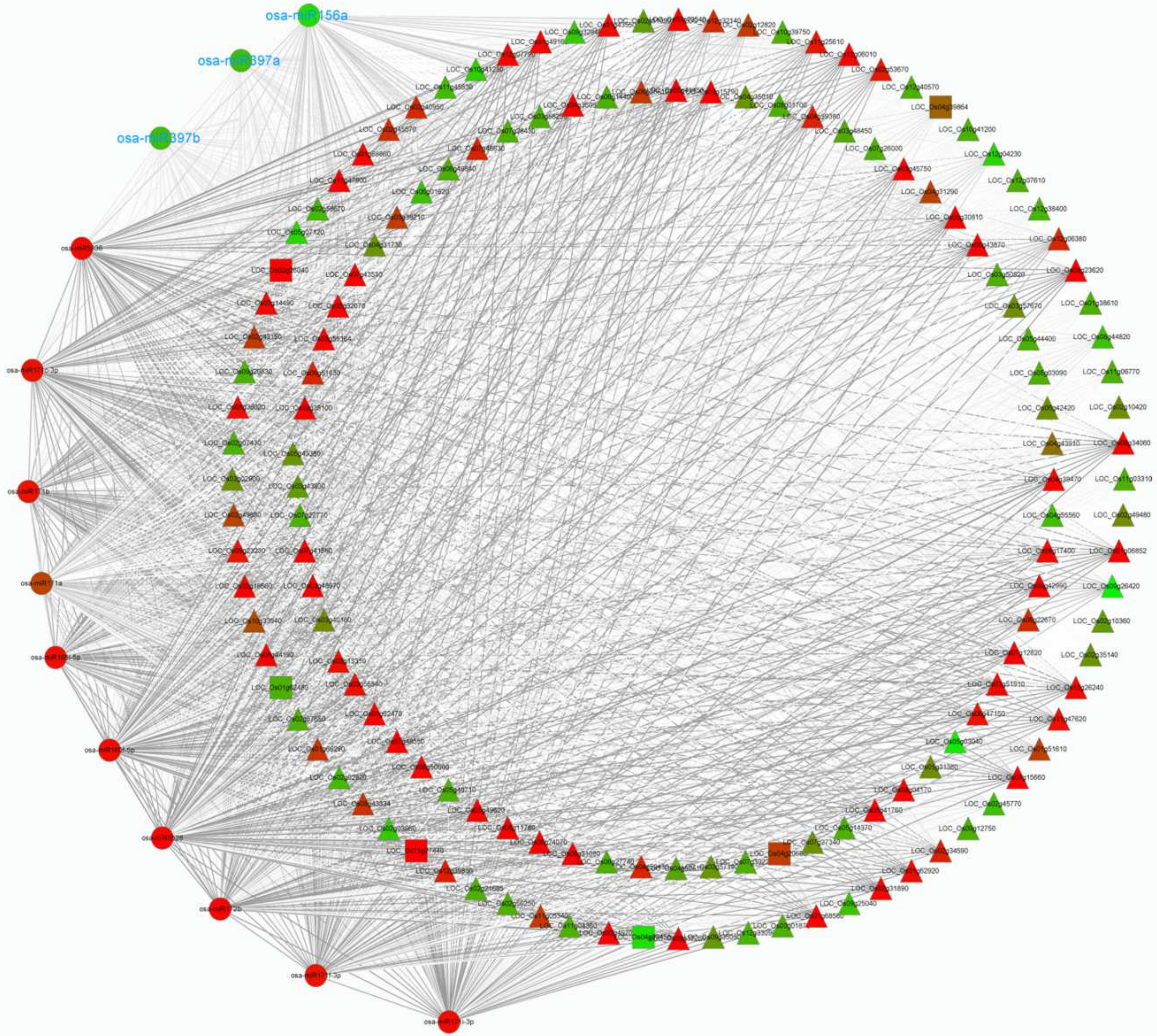

C

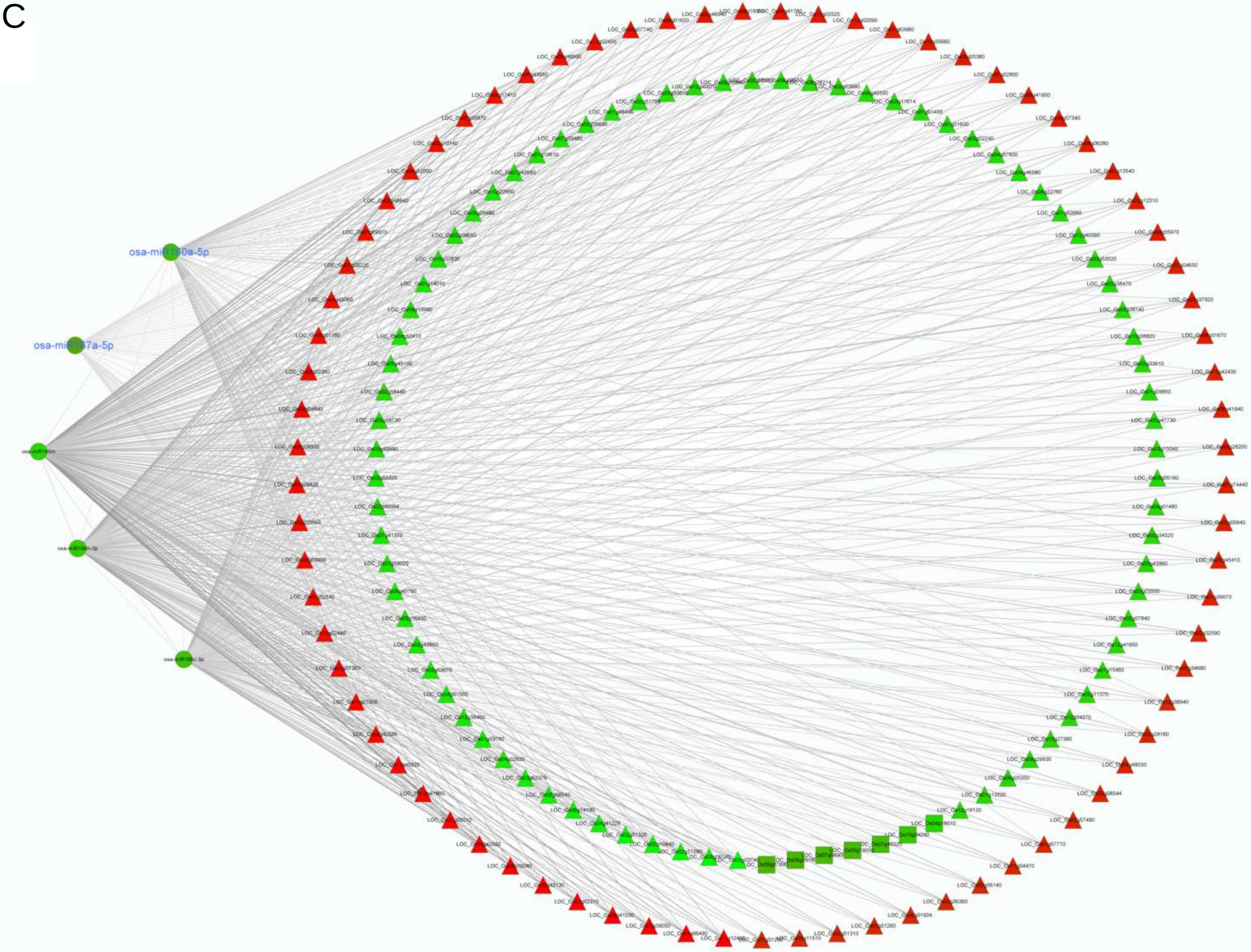

Supplementary Fig. S4 Co-expression networks of candidate yield miRNAs identified by DET with reported yield miRNAs in Taoyuan ultra-high yield rice (A, tiller, B, panicle and C, flag leaf). Rectangles: miRNAs, circles: genes, triangles: TF.

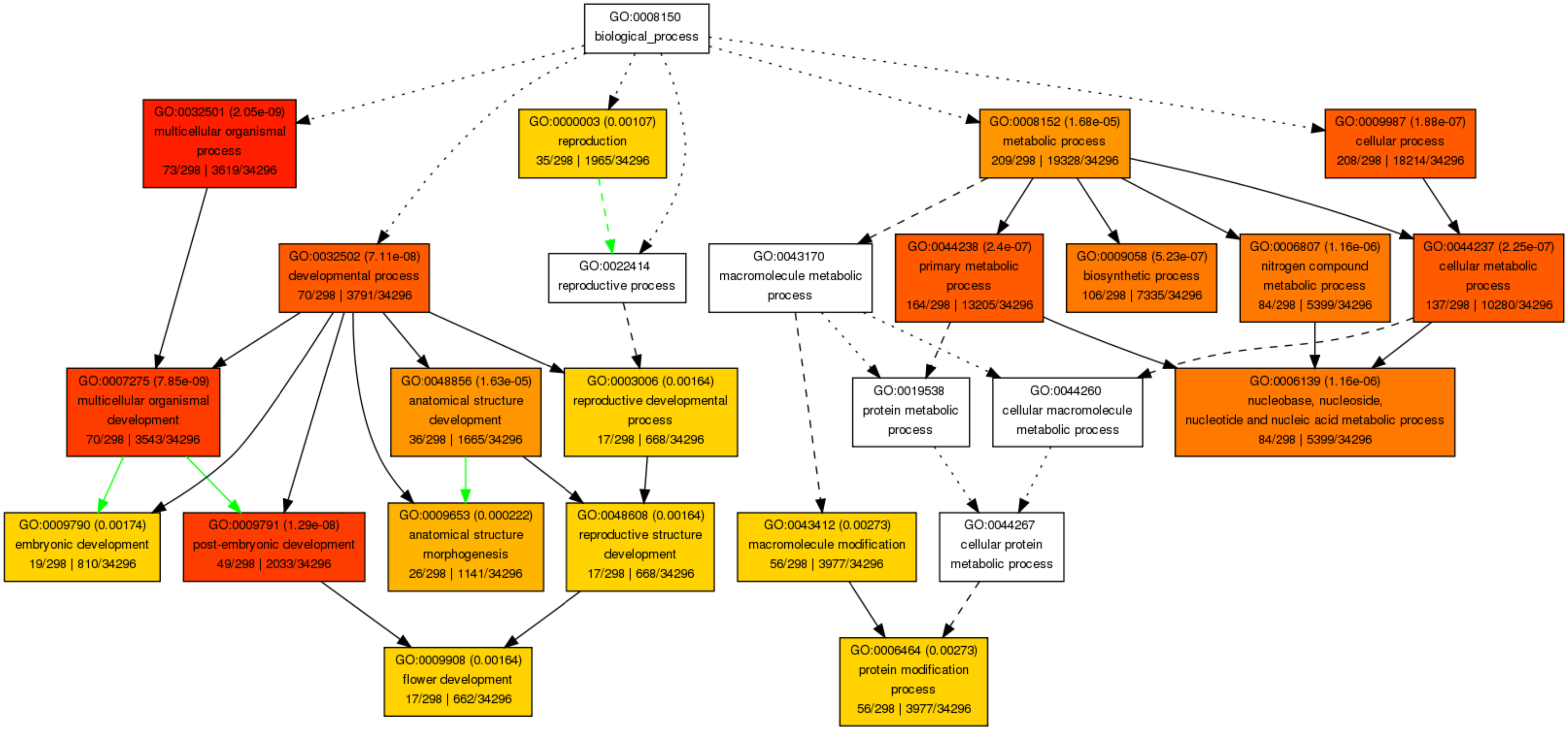

Supplementary Fig. S5 GO enrichment of genes co-regulated with sample specific miRNAs (SmiRNAs) which are identified by differential edge-like transformation (DET) between Taoyuan and Jinghong samples.

Supplementary Table S1 Details of sample-specific miRNAs (SmiRNAs) in different tissues at the key yield stage analyzed using differential edge-like transformation (DET) analysis with their expression levels in each sample.

Rank	miRNA	Grain filling				
		**DAF10** FC (S/I)	**DAF15** FC (S/I)	**DAF21** FC (S/I)	**DAF27** FC (S/I)	**△PCC** (S-I)
1	osa-miR2105	0.000	0.000	0.000	0.090	0.8933
**2**	**osa-miR395n**	**0.000**	**0.000**	**0.000**	**0.090**	**0.8933**
3	osa-miR2096-5p	0.000	0.000	0.090	0.090	0.7594
4	osa-miR1861c	0.140	0.080	0.000	0.000	0.7448
5	osa-miR1862g	0.070	0.080	0.000	0.000	0.7448
**6**	**osa-miR444b**	**0.647**	**2.666**	**1.305**	**3.061**	**0.7290**
**7**	**osa-miR444c**	**0.647**	**2.666**	**1.305**	**3.061**	**0.7290**
8	osa-miR812l	1.577	2.319	2.612	1.054	0.7247
9	osa-miR164b	0.607	0.743	0.287	0.337	0.7205
10	osa-miR164a	0.607	0.743	0.289	0.337	0.7200
11	osa-miR164f	0.608	0.743	0.2870	0.337	0.7205
12	osa-miR812a	2.712	3.160	5.092	1.047	0.7205
13	osa-miR812c	2.419	3.390	3.461	1.233	0.7110
**14**	**osa-miR1432**	**0.385**	**0.643**	**0.231**	**0.171**	**0.6716**
15	osa-miR812e	2.451	3.270	3.544	1.182	0.6605
**16**	**osa-miR2102-5p**	**0.560**	**0.080**	**0.090**	**0.000**	**0.6459**
**17**	**osa-miR166l**	**0.700**	**0.685**	**0.265**	**0.355**	**0.5894**
**18**	**osa-miR166k**	**0.709**	**0.696**	**0.268**	**0.361**	**0.5741**
19	osa-miR1846a-3p	0.539	0.150	0.948	2.000	0.5553
**20**	**osa-miR169a**	**0.898**	**0.353**	**0.278**	**0.065**	**0.5124**

Rank	miRNA	Taoyuan	Jinghong	PCC (Taoyuan)	PCC (Jinghong)	△PCC (Taoyuan-Jinghong)
**Tillers**						
1	osa-miR1428e-3p	1.474	1.477	2.132	0.000	2.133
2	osa-miR1861i	4.069	3.545	2.144	0.031	2.112
3	osa-miR1861f	4.069	3.545	2.144	0.031	2.112
4	osa-miR1861l	4.069	3.545	2.144	0.031	2.112
5	osa-miR1861b	4.069	3.545	2.144	0.031	2.112
6	osa-miR167e-3p	12.913	13.293	2.143	0.042	2.100
7	osa-miR167i-3p	12.913	13.293	2.143	0.042	2.100
8	osa-miR396b-5p	77.481	83.895	2.132	0.033	2.099
9	osa-miR396a-5p	77.539	83.895	2.132	0.033	2.099
10	osa-miR169m	7.194	6.499	2.145	0.056	2.093
11	osa-miR169l	7.194	6.499	2.145	0.056	2.091
12	osa-miR169k	7.194	6.499	2.145	0.056	2.091

13	osa-miR169j	7.194	6.499	2.145	0.056	2.091
14	osa-miR169i-5p.1	7.194	6.499	2.145	0.056	2.091
15	osa-miR396f-3p	24.641	25.240	2.156	0.075	2.081
16	osa-miR3979-3p	14.608	3.770	2.158	0.082	2.077
17	osa-miR1870-5p	92.864	151.110	2.140	0.066	2.074
18	osa-miR167d-3p	2.649	0.410	2.159	0.088	2.071
19	osa-miR396e-5p	10.514	4.015	2.117	0.047	2.070
20	osa-miR396f-5p	5813.221	1200.491	2.117	0.047	2.070
21	osa-miR5825	2.536	1.477	2.162	0.101	2.062
22	osa-miR169g	2.2407	3.2495	2.070	0.020	2.050
23	osa-miR169f.1	2.2407	3.2495	2.070	0.020	2.050
24	osa-miR396d	37.325	33.381	2.141	0.093	2.048
25	osa-miR396g	37.325	33.381	2.141	0.093	2.048
26	osa-miR396h	37.325	33.381	2.141	0.093	2.048
27	osa-miR818d	1.179	0.591	2.166	0.120	2.046
28	osa-miR3979-5p	182.557	231.598	2.107	0.063	2.043
29	osa-miR1871	14.152	20.678	2.108	0.075	2.033
30	osa-miR810b.1	6.84	3.5449	2.166	0.134	2.032
31	osa-miR529b	13.444	5.613	2.169	0.140	2.030
32	osa-miR390-3p	24.765	15.361	2.160	0.133	2.027
33	osa-miR169b	1.533	0.591	2.171	0.147	2.023
34	osa-miR171h	24.589	9.158	2.171	0.150	2.021
35	osa-miR167e-5p	220.589	176.062	2.150	0.131	2.018
36	osa-miR167i-5p	219.882	175.176	2.150	0.132	2.017
37	osa-miR1861m	13.562	4.727	2.172	0.156	2.016
38	osa-miR1861k	13.562	4.727	2.172	0.156	2.016
39	osa-miR1861e	13.562	4.727	2.172	0.156	2.016
40	osa-miR5145	3.007	2.068	2.144	0.132	2.012
**Young panicles**						
1	osa-miR5834	2.105	0.483	1.529	0.034	1.495
2	osa-miR2863c	2.679	0.483	1.539	0.107	1.432
3	osa-miR160f-3p	1.340	0.241	1.539	0.107	1.432
4	osa-miR2879	1.531	0.483	1.506	0.094	1.412
5	osa-miR5528	1.531	0.241	1.543	0.140	1.403
6	osa-miR5488	1.723	0.241	1.546	0.166	1.380
7	osa-miR5499	1.914	0.242	1.547	0.186	1.361
8	osa-miR5143b	3.445	1.448	1.468	0.248	1.220
9	osa-miR5802	1.722	0.724	1.468	0.248	1.220
10	osa-miR172b	1.722	0.724	1.468	0.248	1.220
11	osa-miR2876-3p	2.295	0.01	1.555	0.367	1.188
12	osa-miR5518	1.340	0.01	1.555	0.367	1.188
13	osa-miR5514	3.253	1.448	1.458	0.283	1.174
14	osa-miR160f-5p	9.377	2.413	1.418	0.265	1.153
15	osa-miR171i-5p	3.827	3.137	1.446	0.298	1.148

16	osa-miR5836	8.803	4.102	1.448	0.313	1.135
17	osa-miR166l-5p	13.970	7.722	1.407	0.434	0.973
18	osa-miR5143a	3.445	1.448	1.079	0.137	0.941
19	osa-miR1863c	6.124	4.344	1.230	0.322	0.907
20	osa-miR171f-3p	43.631	30.888	1.343	0.488	0.855
21	osa-miR1846e	0.766	1.207	0.852	0.000	0.852
22	osa-miR171d-3p	44.014	31.370	1.338	0.502	0.830
23	osa-miR171e-3p	43.823	31.370	1.336	0.506	0.830
24	osa-miR167c-3p	2.296	1.448	1.366	0.537	0.829
25	osa-miR5538	1.531	0.965	1.366	0.537	0.829
26	osa-miR171b	55.496	40.057	1.349	0.526	0.823
27	osa-miR172d-3p	8.420	8.204	1.349	0.526	0.823
28	osa-miR5487	2.488	1.690	1.339	0.599	0.739
29	osa-miR1859	1.722	1.207	1.326	0.626	0.700
30	osa-miR1883b	14.544	11.100	0.915	0.315	0.600
31	osa-miR2118i	16.266	12.307	1.293	0.694	0.599
32	osa-miR171a	3.827	2.896	0.838	0.259	0.548
33	osa-miR166k-3p	2036.889	1719.811	1.250	0.702	0.548
34	osa-miR166l-3p	2215.432	1904.654	1.244	0.727	0.517
35	osa-miR5796	18.180	14.720	1.261	0.755	0.506
**Flag leaves**						
1	osa-miR1425-3p	1.911	0.970	1.195	0.495	0.699
2	osa-miR1857-3p	1.528	0.776	1.195	0.495	0.699
3	osa-miR2873a	14.138	7.374	1.187	0.516	0.671
4	osa-miR172d-3p	18.150	11.450	1.061	0.451	0.611
5	osa-miR166m	98.774	282.166	0.706	0.103	0.604
6	osa-miR172a	18.150	11.450	1.033	0.466	0.567
7	osa-miR5513	1.146	0.194	0.853	0.323	0.530
8	osa-miR166d-5p	12.418	23.676	0.531	0.004	0.527
9	osa-miR166h-3p	341.028	891.325	0.603	0.078	0.525
10	osa-miR166j-3p	13818.22	32552.27	0.516	0.014	0.502
11	osa-miR166c-3p	13818.22	32595.74	0.516	0.014	0.502
12	osa-miR2863b	2.866	1.941	1.021	0.578	0.444
13	osa-miR2864.1	3.248	2.135	1.100	0.702	0.397
14	osa-miR166b-3p	7541.59	25320.18	0.574	0.188	0.285
15	osa-miR166f	7548.08	25333.96	0.574	0.188	0.385
16	osa-miR166d-3p	7549.99	25341.53	0.574	0.188	0.385
17	osa-miR166a-3p	7549.80	25341.14	0.574	0.188	0.385
18	osa-miR156l-3p	1.720	1.164	1.086	0.728	0.359
19	osa-miR167a-3p	3.63	0.970	0.730	0.385	0.345
20	osa-miR827	20.824	15.525	0.853	0.534	0.320
21	osa-miR3982-3p	2.484	1.747	1.069	0.760	0.309
22	osa-miR812m	2.866	4.075	0.338	0.032	0.305
23	osa-miR167h-3p	13.374	13.373	1.000	0.713	0.287

24	osa-miR1425-5p	1425.057	623.326	0.775	0.516	0.259
25	osa-miR395i	1.528	0.388	0.675	0.420	0.255
26	osa-miR395j	1.528	0.388	0.675	0.420	0.255
27	osa-miR395k	1.528	0.388	0.675	0.420	0.255
28	osa-miR1860-3p	2.866	1.358	0.575	0.325	0.250
29	osa-miR156c-3p	0.573	1.552	0.328	0.086	0.242
30	osa-miR156g-3p	0.573	1.552	0.328	0.086	0.242
31	osa-miR395h	1.528	0.388	0.634	0.394	0.240
32	osa-miR395m	1.528	0.388	0.634	0.394	0.240
33	osa-miR535-5p	15.475	33.573	0.668	0.437	0.230
34	osa-miR166j-5p	97.054	77.042	0.767	0.561	0.206

Note: FC, fold change.

Supplementary Table S2 The differentially expressed miRNAs (DEmiRNAs) in tillers, young panicles and flag leaves of IR64 rice planted at Taoyuan and Jinghong.

miRNA	Taoyuan	Jinghong	Fold-change	*P*-value
**Tillers**				
osa-miR159a.1	69.9919	184.3334	1.397058	0.0000
osa-miR159b	70.0508	184.3334	1.395844	0.0000
osa-miR160a-3p	2.0048	0.2954	-2.76272	0.0234
osa-miR160b-3p	2.0048	0.2954	-2.76272	0.0234
osa-miR164c	0.059	1.477	4.645811	0.0003
osa-miR166e-3p	5.6017	2.3632	-1.24512	0.0127
osa-miR169c	7.5476	1.477	-2.35335	0.0000
osa-miR169e	1.651	0.01	-7.3672	0.0102
osa-miR169i-5p.2	0.5897	6.4989	3.462142	0.0000
osa-miR169n	1.8279	0.2954	-2.62945	0.0374
osa-miR169o	1.8279	0.2954	-2.62945	0.0374
osa-miR169r-3p	1.0614	5.6127	2.402727	0.0000
osa-miR169r-5p	0.7665	5.6127	2.872337	0.0000
osa-miR171h	24.5885	9.1576	-1.42494	0.0000
osa-miR171i-3p	2.0638	4.1357	1.002828	0.0273
osa-miR172d-3p	0.3538	1.1816	1.739736	0.0487
osa-miR172d-5p	0.2948	1.477	2.324861	0.0091
osa-miR1846e	0.2948	1.1816	2.002933	0.0307
osa-miR1861e	13.562	4.7265	-1.52073	0.0000
osa-miR1861h	22.4068	3.2495	-2.78565	0.0000
osa-miR1861j	22.4068	3.2495	-2.78565	0.0000
osa-miR1861k	13.562	4.7265	-1.52073	0.0000
osa-miR1861m	13.562	4.7265	-1.52073	0.0000
osa-miR1868	13.4441	48.1512	1.840599	0.0000
osa-miR1883a	18.1024	7.976	-1.18244	0.0000
osa-miR2880	14.4465	2.3632	-2.61191	0.0000
osa-miR319a-3p	6.722	20.6784	1.621162	0.0000
osa-miR393a	4.8352	0.5908	-3.03283	0.0001
osa-miR393b-5p	3.8917	1.1816	-1.71966	0.0103
osa-miR395b	0.4717	2.6587	2.494779	0.0004
osa-miR395d	0.3538	2.9541	3.061713	0.0000
osa-miR395e	0.4128	2.6587	2.687206	0.0002
osa-miR395g	0.3538	2.6587	2.909715	0.0001
osa-miR395h	0.2948	2.3632	3.002933	0.0002
osa-miR395i	0.2948	2.6587	3.172913	0.0000
osa-miR395j	0.2948	2.6587	3.172913	0.0000

osa-miR395k	0.2948	2.6587	3.172913	0.0000
osa-miR395l	0.2948	2.6587	3.172913	0.0000
osa-miR395m	0.2948	2.3632	3.002933	0.0002
osa-miR395n	0.2948	2.6587	3.172913	0.0000
osa-miR395p	0.2948	2.6587	3.172913	0.0000
osa-miR395q	0.2948	2.9541	3.32491	0.0000
osa-miR395r	0.2948	2.9541	3.32491	0.0000
osa-miR395s	0.3538	2.6587	2.909715	0.0001
osa-miR395y	0.3538	2.6587	2.909715	0.0001
osa-miR397a	34.5537	69.7158	1.012645	0.0000
osa-miR397b	361.5755	904.829	1.323348	0.0000
osa-miR398a	1.8279	0.01	-7.51404	0.0059
osa-miR398b	2.8893	10.6346	1.879974	0.0000
osa-miR399d	7.4296	2.9541	-1.33057	0.0022
osa-miR5156	0.5897	2.9541	2.324666	0.0004
osa-miR528-3p	0.4717	9.7484	4.369224	0.0000
osa-miR528-5p	0.4128	5.0219	3.604718	0.0000
osa-miR529b	13.4441	5.6127	-1.26021	0.0001
osa-miR531a	5.8965	2.0678	-1.51176	0.0030
osa-miR531b	5.8965	2.0678	-1.51176	0.0030
osa-miR531c	5.8965	2.0678	-1.51176	0.0030
osa-miR5539a	0.3538	2.0678	2.547091	0.0014
osa-miR5539b	0.3538	2.0678	2.547091	0.0014
osa-miR812c	5.7786	1.477	-1.96805	0.0005
osa-miR812d	2.9483	0.8862	-1.73418	0.0287
osa-miR812e	7.5476	2.0678	-1.86792	0.0001
osa-miR812v	3.3021	1.1816	-1.48264	0.0362
**Young panicles**				
osa-miR160f-5p	9.3769	2.4131	-1.95822	0.0000
osa-miR167a-3p	2.105	4.8262	1.197067	0.0241
osa-miR171c-5p	4.4014	8.9284	1.020439	0.0068
osa-miR171d-5p	5.9323	12.5481	1.080805	0.0008
osa-miR1850.1	3.2532	8.2045	1.334556	0.0013
osa-miR1880	1.3396	4.5849	1.775088	0.0033
osa-miR2106	1.7223	6.7567	1.971982	0.0001
osa-miR2863c	2.6791	0.4826	-2.47285	0.0098
osa-miR2871a-3p	7.6546	3.137	-1.28694	0.0035
osa-miR2871a-5p	24.4947	8.4458	-1.53616	0.0000
osa-miR2871b	7.6546	3.137	-1.28694	0.0035
osa-miR2876-3p	2.2964	0.01	-7.84323	0.0010
osa-miR2878-5p	4.21	1.2065	-1.80299	0.0065
osa-miR396c-3p	2.4877	5.3088	1.093573	0.0277
osa-miR408-5p	0.01	1.2065	6.914684	0.0150
osa-miR444d.3	1.7223	0.2413	-2.83544	0.0316

osa-miR528-5p	5.3582	13.5133	1.33456	0.0000
osa-miR529a	20.4761	81.3212	1.989691	0.0000
osa-miR531a	1.1482	4.1023	1.837059	0.0044
osa-miR531b	1.1482	4.1023	1.837059	0.0044
osa-miR531c	1.1482	4.1023	1.837059	0.0044
osa-miR5486	0.3827	2.1718	2.504605	0.0136
osa-miR5488	1.7223	0.2413	-2.83544	0.0316
osa-miR5499	1.9136	0.2413	-2.98739	0.0190
osa-miR5505	1.1482	4.1023	1.837059	0.0044
osa-miR5513	1.1482	0.01	-6.84323	0.0336
osa-miR5518	1.3396	0.01	-7.06566	0.0187
osa-miR5519	0.7655	3.8609	2.334463	0.0013
osa-miR5834	2.105	0.4826	-2.12492	0.0391
osa-miR5836	8.8028	4.1023	-1.10153	0.0055
osa-miR815b	0.01	1.2065	6.914684	0.0150
**Flag leaves**				
osa-miR1425-5p	1425.057	623.3257	-1.19296	0.0000
osa-miR1432-5p	83.4897	195.42	1.226908	3.85E-54
osa-miR159c	2.1016	4.6575	1.148068	0.0256
osa-miR159d	2.1016	4.6575	1.148068	0.0256
osa-miR159e	2.1016	4.6575	1.148068	0.0256
osa-miR160e-5p	2.2926	4.6575	1.022571	0.0420
osa-miR164d	0.5732	2.9109	2.344355	0.0040
osa-miR166a-3p	7549.801	25341.14	1.746971	0.0000
osa-miR166b-3p	7541.585	25320.18	1.747348	0.0000
osa-miR166c-3p	13836.37	32595.74	1.236219	0.0000
osa-miR166d-3p	7549.992	25341.53	1.746957	0.0000
osa-miR166e-3p	1.1463	3.105	1.437609	0.0316
osa-miR166f	7548.081	25333.96	1.746891	0.0000
osa-miR166g-3p	1694.249	6291.864	1.892842	0.0000
osa-miR166h-3p	341.0278	891.3247	1.386062	0.0000
osa-miR166i-3p	36.682	84.4168	1.202458	0.0000
osa-miR166j-3p	13818.22	32552.27	1.236187	0.0000
osa-miR166k-3p	118.0701	245.6819	1.057148	0.0000
osa-miR166l-3p	132.208	298.6607	1.175698	0.0000
osa-miR166m	98.7739	282.1655	1.51434	0.0000
osa-miR167a-3p	3.63	0.9703	-1.90347	0.0046
osa-miR168a-3p	2994.931	6580.822	1.135745	0.0000
osa-miR1846d-3p	1.7195	4.4634	1.376154	0.0119
osa-miR1846d-5p	0.5732	2.1347	1.896923	0.0326
osa-miR1865-3p	2.8658	0.5822	-2.29935	0.0049
osa-miR2871a-5p	58.653	29.3033	-1.00114	0.0000
osa-miR2878-5p	3.2479	0.5822	-2.47992	0.0017
osa-miR2880	1.1463	3.8812	1.759518	0.0052

osa-miR397a	148.4474	782.2622	2.3977	0.0000
osa-miR397b	1499.185	8553.069	2.512264	0.0000
osa-miR398a	3.63	0.3881	-3.22547	0.0001
osa-miR398b	134.8827	1467.3	3.443386	0.0000
osa-miR408-3p	265.1801	2552.298	3.266752	0.0000
osa-miR408-5p	4.2031	26.7805	2.671657	0.0000
osa-miR5083	0.1911	1.3584	2.829509	0.0369
osa-miR528-3p	61.7098	291.0923	2.237905	0.0000
osa-miR528-5p	52.1572	218.5133	2.066783	0.0000
osa-miR535-5p	15.4752	33.5727	1.117331	0.0000
osa-miR7694-5p	1.3374	0.1941	-2.78456	0.0413

Supplementary Table S3 A set of 150 sequencing screened yield related miRNAs from literatures.

ID	miRNA	Target
1	osa-miR1320-3p	LOC_Os03g13800; LOC_Os05g38590; LOC_Os08g19610; LOC_Os05g31230; LOC_Os11g02464; LOC_Os12g02390; LOC_Os06g16919; LOC_Os06g03600; LOC_Os01g48250; LOC_Os06g09170; LOC_Os03g37920
2	osa-miR1432-3p	LOC_Os10g30540; LOC_Os04g08350; LOC_Os06g40940
3	osa-miR1435	LOC_Os03g42280; LOC_Os04g44354; LOC_Os04g42444; LOC_Os08g29260; LOC_Os07g13020; LOC_Os04g41238; LOC_Os05g22910; LOC_Os04g50204; LOC_Os09g07200; LOC_Os06g43910; LOC_Os12g16060; LOC_Os03g48320; LOC_Os07g36080
4	**osa-miR156a**	LOC_Os11g30370; LOC_Os09g32944; **LOC_Os09g31438**; LOC_Os06g45310; LOC_Os02g07780; LOC_Os08g41940; **LOC_Os08g39890**; LOC_Os01g69830; LOC_Os07g32170; LOC_Os02g04680; LOC_Os09g03670; LOC_Os09g33820
5	osa-miR156b-3p	LOC_Os03g26650
6	osa-miR156b-5p	LOC_Os11g30370; LOC_Os09g32944; LOC_Os09g31438; LOC_Os06g45310; LOC_Os02g07780; LOC_Os08g41940; LOC_Os08g39890; LOC_Os01g69830; LOC_Os02g07780; LOC_Os07g32170; LOC_Os02g04680; LOC_Os09g03670; LOC_Os09g33820
7	osa-miR156c-3p	LOC_Os07g40390; LOC_Os08g04180; LOC_Os02g04490; LOC_Os04g37670 LOC_Os07g15370
8	osa-miR156c-5p	LOC_Os11g30370; LOC_Os09g32944; LOC_Os09g31438; LOC_Os06g45310; LOC_Os02g07780; LOC_Os08g41940; LOC_Os08g39890; LOC_Os01g69830; LOC_Os02g07780; LOC_Os07g32170; LOC_Os02g04680; LOC_Os02g04680; LOC_Os09g03670; LOC_Os09g33820
9	osa-miR156d	LOC_Os11g30370; LOC_Os09g32944; LOC_Os09g31438; LOC_Os06g45310; LOC_Os02g07780; LOC_Os08g41940; LOC_Os08g39890; LOC_Os01g69830; LOC_Os02g07780; LOC_Os07g32170; LOC_Os02g04680; LOC_Os09g03670; LOC_Os09g33820
10	osa-miR156f-3p	LOC_Os07g37580; LOC_Os07g40390; LOC_Os01g03170; LOC_Os09g36670 LOC_Os07g31340; LOC_Os05g25210; LOC_Os07g49270
11	**osa-miR156f-5p**	LOC_Os11g30370; LOC_Os09g32944; LOC_Os09g31438; LOC_Os06g45310; LOC_Os02g07780; LOC_Os08g41940; **LOC_Os08g39890**; LOC_Os01g69830; LOC_Os02g07780; LOC_Os07g32170; LOC_Os02g04680; LOC_Os09g03670; LOC_Os09g33820
12	osa-miR156g-3p	LOC_Os07g40390; LOC_Os08g04180; LOC_Os02g04490; LOC_Os04g37670 LOC_Os07g15370
13	osa-miR156g-5p	LOC_Os11g30370; LOC_Os09g32944; LOC_Os09g31438; LOC_Os06g45310; LOC_Os02g07780; LOC_Os08g41940; LOC_Os08g39890; LOC_Os01g69830; LOC_Os02g07780; LOC_Os07g32170; LOC_Os02g04680; LOC_Os09g03670; LOC_Os09g33820
14	osa-miR156h-3p	LOC_Os07g37580; LOC_Os07g40390; LOC_Os01g03170; LOC_Os09g36670; LOC_Os07g31340; LOC_Os05g25210; LOC_Os07g49270
15	osa-miR156h-5p	LOC_Os11g30370; LOC_Os09g32944; LOC_Os09g31438; LOC_Os06g45310; LOC_Os02g07780; LOC_Os08g41940; LOC_Os08g39890; LOC_Os01g69830; LOC_Os02g07780; LOC_Os07g32170; LOC_Os02g04680; LOC_Os09g03670;

		LOC_Os09g33820
16	osa-miR156i	LOC_Os11g30370; LOC_Os09g32944; LOC_Os09g31438; LOC_Os06g45310; LOC_Os02g07780; LOC_Os08g41940; LOC_Os08g39890; LOC_Os01g69830; LOC_Os02g07780; LOC_Os07g32170; LOC_Os02g04680; LOC_Os09g03670; LOC_Os09g33820
17	osa-miR156j-3p	LOC_Os05g13940; LOC_Os04g34890
18	osa-miR156j-5p	LOC_Os11g30370; LOC_Os09g32944; LOC_Os09g31438; LOC_Os06g45310; LOC_Os02g07780; LOC_Os08g41940; LOC_Os08g39890; LOC_Os01g69830; LOC_Os02g07780; LOC_Os07g32170; LOC_Os02g04680; LOC_Os09g03670; LOC_Os09g33820
19	**osa-miR159a.1**	LOC_Os01g12700; LOC_Os05g41166; LOC_Os09g36650; LOC_Os06g40330 LOC_Os05g42240; **LOC_Os01g59660;** LOC_Os01g11430; LOC_Os03g21380 LOC_Os03g47949
20	osa-miR159a.2	LOC_Os03g02240; LOC_Os10g40920; LOC_Os03g28310; LOC_Os08g10800 LOC_Os02g05720; LOC_Os12g24550
21	osa-miR160a-3p	LOC_Os02g56000; LOC_Os02g49240; LOC_Os08g43570; LOC_Os11g38140 LOC_Os07g37130
22	osa-miR160a-5p	*LOC_Os10g33940*; LOC_Os04g43910; LOC_Os09g29160; *LOC_Os04g59430* LOC_Os02g44760
23	osa-miR160b-3p	LOC_Os02g56000; LOC_Os02g49240; LOC_Os08g43570; LOC_Os11g38140 LOC_Os07g37130
24	osa-miR160b-5p	*LOC_Os10g33940*; LOC_Os04g43910; LOC_Os09g29160; *LOC_Os04g59430* LOC_Os02g44760
25	osa-miR160c-5p	*LOC_Os10g33940*; *LOC_Os04g43910*; LOC_Os09g29160; *LOC_Os04g59430* LOC_Os02g44760
26	osa-miR160d-3p	LOC_Os05g18840; LOC_Os10g29020
27	osa-miR160d-5p	*LOC_Os10g33940* ; *LOC_Os04g43910*; LOC_Os09g29160; LOC_Os04g59430 LOC_Os02g44760
28	osa-miR160e-3p	LOC_Os06g37480; LOC_Os09g39430
29	osa-miR160e-5p	*LOC_Os10g33940; LOC_Os04g43910*; LOC_Os09g29160; *LOC_Os04g59430* LOC_Os02g44760
		LOC_Os03g01890; LOC_Os10g33960; LOC_Os12g41860; LOC_Os03g43930;
30	osa-miR160f-3p	LOC_Os03g01890; LOC_Os03g44835; LOC_Os04g23200; LOC_Os01g33740; LOC_Os03g35380; LOC_Os07g08900; LOC_Os08g34740
31	osa-miR160f-5p	*LOC_Os04g59430*; *LOC_Os10g33940*; *LOC_Os04g43910*; LOC_Os09g29160 LOC_Os09g25200; LOC_Os02g37790; LOC_Os02g26040; LOC_Os02g49930 LOC_Os07g29360
32	osa-miR164e	LOC_Os03g47310; LOC_Os06g23650; LOC_Os06g46270; *LOC_Os12g41680*; LOC_Os07g12620; LOC_Os06g05760; LOC_Os09g32920; LOC_Os10g42090 LOC_Os05g04630; LOC_Os04g40970; LOC_Os01g04950; LOC_Os02g18690
33	osa-miR166a-3p	*LOC_Os03g01890;* LOC_Os10g33960; *LOC_Os12g41860;* *LOC_Os03g43930* LOC_Os03g01890; LOC_Os03g44835; LOC_Os04g23200; LOC_Os01g33740; LOC_Os03g35380; LOC_Os07g08900; LOC_Os08g34740
34	osa-miR166a-5p	LOC_Os09g08440; LOC_Os12g24410; LOC_Os09g25520; LOC_Os06g36960;

		LOC_Os03g60820; LOC_Os03g14260; LOC_Os08g14940; LOC_Os07g07910; LOC_Os06g01304; LOC_Os05g1226
35	osa-miR166b-3p	LOC_Os03g01890; LOC_Os10g33960; LOC_Os12g41860; LOC_Os03g43930; LOC_Os10g33960; LOC_Os03g01890; LOC_Os10g33960; LOC_Os03g44835; LOC_Os04g23200; LOC_Os01g33740; LOC_Os03g35380; LOC_Os07g08900; LOC_Os08g34740
36	osa-miR166b-5p	LOC_Os10g26730; LOC_Os04g04410; LOC_Os03g12480; LOC_Os06g42410; LOC_Os05g39490; LOC_Os03g03610
37	osa-miR166c-3p	LOC_Os03g01890; LOC_Os10g33960; LOC_Os12g41860; LOC_Os03g43930; LOC_Os10g33960; LOC_Os03g01890; LOC_Os10g33960; LOC_Os03g44835; LOC_Os04g23200; LOC_Os01g33740; LOC_Os03g35380; LOC_Os07g08900; LOC_Os08g34740
38	osa-miR166c-5p	LOC_Os01g42520; LOC_Os03g14260; LOC_Os03g60820; LOC_Os07g07910
39	osa-miR166d-3p	LOC_Os03g01890; LOC_Os10g33960; LOC_Os12g41860; LOC_Os03g43930; LOC_Os10g33960; LOC_Os03g01890; LOC_Os10g33960; LOC_Os03g44835; LOC_Os04g23200; LOC_Os01g33740; LOC_Os03g35380; LOC_Os07g08900; LOC_Os08g34740
40	osa-miR166d-5p	LOC_Os10g26730
41	osa-miR166f	LOC_Os03g01890; LOC_Os10g33960; LOC_Os12g41860; LOC_Os03g43930; LOC_Os10g33960; LOC_Os03g01890; LOC_Os10g33960; LOC_Os03g44835; LOC_Os04g23200; LOC_Os01g33740; LOC_Os03g35380; LOC_Os07g08900; LOC_Os08g34740
42	osa-miR167d-3p	LOC_Os11g02520; LOC_Os12g02440; LOC_Os02g12810; LOC_Os02g21910; LOC_Os05g07980; LOC_Os03g62790; LOC_Os03g62790; LOC_Os01g10780; LOC_Os01g22480
43	osa-miR167d-5p	LOC_Os07g29820; LOC_Os10g37590; LOC_Os01g63290; LOC_Os09g37890; LOC_Os06g03830; LOC_Os07g49320; LOC_Os05g19050; LOC_Os04g18010
44	osa-miR167e-3p	LOC_Os08g12760; LOC_Os10g25750; LOC_Os03g31180; LOC_Os02g18660
45	osa-miR167f	LOC_Os07g29820; LOC_Os10g37590; LOC_Os01g63290; LOC_Os09g37890; LOC_Os06g03830; LOC_Os07g49320; LOC_Os05g19050; LOC_Os04g18010
46	osa-miR167h-3p	LOC_Os01g20900; LOC_Os03g25950; LOC_Os06g13560; LOC_Os01g74610; LOC_Os02g55020; LOC_Os01g41565; LOC_Os02g52470; LOC_Os02g52470
47	osa-miR167h-5p	LOC_Os07g29820; LOC_Os10g37590; LOC_Os01g63290; LOC_Os09g37890; LOC_Os06g03830; LOC_Os07g49320; LOC_Os05g19050; LOC_Os04g18010
48	osa-miR167i-3p	LOC_Os08g12760; LOC_Os10g25750; LOC_Os03g31180; LOC_Os02g18660
49	osa-miR167j	LOC_Os07g29820; LOC_Os10g37590; LOC_Os01g63290; LOC_Os09g37890; LOC_Os06g03830; LOC_Os07g49320; LOC_Os05g19050; LOC_Os04g18010
50	osa-miR168a-3p	LOC_Os06g44034; LOC_Os12g20370
51	osa-miR168a-5p	LOC_Os02g47690; LOC_Os01g53160; LOC_Os11g44860; LOC_Os03g48180 LOC_Os02g58490
52	osa-miR169b	LOC_Os10g20990; LOC_Os03g07880; LOC_Os07g41720; LOC_Os12g42400 LOC_Os03g29760; LOC_Os02g53620; LOC_Os11g14570; LOC_Os01g41650
53	osa-miR169g	LOC_Os02g53620; LOC_Os12g42400; LOC_Os03g48970; LOC_Os03g07880; LOC_Os07g41720; LOC_Os03g29760; LOC_Os07g28560; LOC_Os06g13920

54	osa-miR169h	LOC_Os02g53620; LOC_Os03g48970; LOC_Os12g42400; LOC_Os03g07880; LOC_Os03g07880; LOC_Os07g41720; LOC_Os03g29760; LOC_Os07g28560; LOC_Os06g13920
55	osa-miR169i-3p	LOC_Os07g16360; LOC_Os11g38610; LOC_Os02g05230; LOC_Os06g47170 LOC_Os02g51260
56	osa-miR169i-5p.1	LOC_Os02g53620; LOC_Os03g48970; LOC_Os12g42400; LOC_Os03g07880; LOC_Os07g41720; LOC_Os03g29760; LOC_Os03g29760; LOC_Os07g28560; LOC_Os06g13920
57	osa-miR169i-5p.2	LOC_Os11g34110; LOC_Os02g03220; LOC_Os02g57370; LOC_Os05g40720 LOC_Os11g36180
58	osa-miR169j	LOC_Os02g53620; LOC_Os03g48970; LOC_Os12g42400; LOC_Os03g07880; LOC_Os07g41720; LOC_Os03g29760; LOC_Os07g28560; LOC_Os06g13920
59	osa-miR169k	LOC_Os02g53620; LOC_Os03g48970; LOC_Os12g42400; LOC_Os03g07880; LOC_Os07g41720; LOC_Os03g29760; LOC_Os07g28560; LOC_Os06g13920
60	osa-miR169l	LOC_Os02g53620; LOC_Os03g48970; LOC_Os12g42400; LOC_Os03g07880; LOC_Os07g41720; LOC_Os03g29760; LOC_Os07g28560; LOC_Os06g13920;
61	osa-miR169m	LOC_Os02g53620; LOC_Os03g48970; LOC_Os12g42400; LOC_Os03g07880; LOC_Os07g41720; LOC_Os03g29760; LOC_Os07g28560; LOC_Os06g13920;
62	**osa-miR171a**	**LOC_Os02g44360;** LOC_Os06g01620; LOC_Os02g19990
63	osa-miR171b	LOC_Os06g01620; LOC_Os02g44360; LOC_Os09g38330; LOC_Os05g34460; LOC_Os06g02304
64	osa-miR171i-3p	LOC_Os02g44360; LOC_Os06g01620; LOC_Os12g18080; LOC_Os01g60000; LOC_Os11g27440; LOC_Os02g19990
65	osa-miR171i-5p	LOC_Os10g27480; LOC_Os03g06890; LOC_Os07g06770
66	**osa-miR172a**	**LOC_Os05g03040; LOC_Os07g13170;** LOC_Os05g03040; LOC_Os04g55560; LOC_Os02g56320; LOC_Os03g57070; LOC_Os03g13370; LOC_Os05g23720; LOC_Os12g37780; LOC_Os04g58720; LOC_Os01g52120; LOC_Os02g55290; LOC_Os03g47650; LOC_Os12g09570; LOC_Os10g36250; LOC_Os10g41030
67	osa-miR172b	LOC_Os05g03040; LOC_Os07g13170; LOC_Os05g03040; LOC_Os04g55560; LOC_Os01g39810; LOC_Os05g45980; LOC_Os04g36054; LOC_Os05g07070; LOC_Os05g07070; LOC_Os05g26926; LOC_Os05g26902; LOC_Os03g44420; LOC_Os02g56320; LOC_Os12g07800; LOC_Os11g40120; LOC_Os04g54190; LOC_Os10g31240
68	osa-miR172c	LOC_Os05g03040; LOC_Os07g13170; LOC_Os05g03040; LOC_Os05g03040; LOC_Os04g55560; LOC_Os08g39630; LOC_Os08g26870; LOC_Os11g23110; LOC_Os04g02230; LOC_Os04g28750; LOC_Os06g49700; LOC_Os01g67970
69	osa-miR172d-3p	LOC_Os05g03040; LOC_Os07g13170; LOC_Os05g03040; LOC_Os04g55560; LOC_Os02g56320; LOC_Os03g57070; LOC_Os03g13370; LOC_Os05g23720; LOC_Os12g37780; LOC_Os04g58720; LOC_Os01g52120; LOC_Os02g55290; LOC_Os03g47650; LOC_Os12g09570; LOC_Os10g36250; LOC_Os10g41030;
70	osa-miR172d-5p	LOC_Os01g56790; LOC_Os07g04860; LOC_Os06g06050; LOC_Os03g51030; LOC_Os03g19590; LOC_Os05g03174; LOC_Os03g60410; LOC_Os05g03174; LOC_Os01g59180; LOC_Os05g32430
71	osa-miR1850.1	LOC_Os10g36650; LOC_Os03g46440; LOC_Os09g24820; LOC_Os04g33510;

		LOC_Os04g47410; LOC_Os03g01216; LOC_Os03g63260; LOC_Os08g06478; LOC_Os02g48140; LOC_Os08g04650; LOC_Os04g02070; LOC_Os02g05244; LOC_Os06g49250; LOC_Os10g35810
72	osa-miR1850.2	LOC_Os07g07530; LOC_Os04g39160; LOC_Os07g03100; LOC_Os03g25110; LOC_Os01g17470
73	osa-miR1850.3	LOC_Os12g06610; LOC_Os03g39690; LOC_Os08g43580
74	**osa-miR1861b**	**LOC_Os01g63810;** LOC_Os05g51790
75	osa-miR1861e	LOC_Os01g63810; LOC_Os06g44970; LOC_Os03g18710
76	osa-miR1861f	LOC_Os01g63810; LOC_Os05g51790
77	osa-miR1861g	LOC_Os08g32060
78	osa-miR1861i	LOC_Os01g63810; LOC_Os01g63810; LOC_Os05g51790
79	osa-miR1861k	LOC_Os01g63810; LOC_Os06g44970; LOC_Os03g18710
80	osa-miR1861l	LOC_Os01g63810; LOC_Os05g51790
81	osa-miR1861m	LOC_Os01g63810; LOC_Os01g63810; LOC_Os06g44970; LOC_Os03g18710
82	osa-miR1862d	LOC_Os10g40960; LOC_Os02g30730; LOC_Os02g39070; LOC_Os03g52280; LOC_Os05g25890; LOC_Os01g53390; LOC_Os03g17634; LOC_Os07g23340; LOC_Os06g41930; LOC_Os01g09330
83	osa-miR1862e	LOC_Os02g30730; LOC_Os03g51230; LOC_Os03g52280; LOC_Os03g01920; LOC_Os06g50070; LOC_Os11g39209; LOC_Os10g12390; LOC_Os06g43660
84	osa-miR1874-3p	LOC_Os02g20950
85	osa-miR1874-5p	LOC_Os05g18774; LOC_Os10g04290; LOC_Os08g28410
86	osa-miR1881	LOC_Os03g15590; LOC_Os08g43120; LOC_Os06g10130; LOC_Os11g47030
87	osa-miR2055	LOC_Os10g07090; LOC_Os01g65080; LOC_Os06g28980; LOC_Os02g17470; LOC_Os05g27910; LOC_Os09g39690; LOC_Os11g01074; LOC_Os12g02260; LOC_Os12g02260; LOC_Os04g53720; LOC_Os02g19890; LOC_Os08g39030
88	osa-miR2863b	LOC_Os04g58580; LOC_Os09g17560; LOC_Os04g58580; LOC_Os01g49000; LOC_Os04g01500; LOC_Os04g53830
89	osa-miR2871b	LOC_Os04g56710; LOC_Os12g31460; LOC_Os02g16020
90	osa-miR390-5p	*LOC_Os02g10100*; LOC_Os03g51040; LOC_Os04g29990; LOC_Os11g36140; LOC_Os04g30060; LOC_Os05g34270; LOC_Os05g33160; LOC_Os04g30330; LOC_Os04g45170; LOC_Os04g29960; LOC_Os01g33110; LOC_Os06g03970; LOC_Os04g46700
91	**osa-miR393a**	**LOC_Os05g05800; LOC_Os04g32460;** LOC_Os04g32460; LOC_Os05g41010 LOC_Os03g52320; LOC_Os03g36080; LOC_Os07g48620; LOC_Os02g06260
92	osa-miR396a-3p	LOC_Os08g45210; LOC_Os02g13270; LOC_Os08g27040; LOC_Os11g47130
93	osa-miR396a-5p	LOC_Os01g32750; LOC_Os04g57050; LOC_Os04g57070; LOC_Os06g28060; LOC_Os01g52640; LOC_Os09g29584; LOC_Os11g45410; LOC_Os06g10310; LOC_Os06g02560; LOC_Os04g51190; LOC_Os07g40820; LOC_Os06g02560; LOC_Os03g51970; LOC_Os12g29980; LOC_Os02g53690; LOC_Os03g47140; LOC_Os11g35030; LOC_Os01g08560; LOC_Os01g08560; LOC_Os04g56740; LOC_Os12g10180; LOC_Os06g19380; LOC_Os05g10580; LOC_Os10g32680; LOC_Os10g32680; LOC_Os04g13100; LOC_Os04g24190
94	osa-miR396b-3p	LOC_Os08g45210; LOC_Os02g13270; LOC_Os08g27040; LOC_Os11g47130
95	**osa-miR396b-5p**	LOC_Os01g32750; LOC_Os04g57050; LOC_Os04g57070; LOC_Os06g28060;

		LOC_Os01g52640; LOC_Os09g29584; LOC_Os11g45410; LOC_Os06g10310; LOC_Os06g02560; LOC_Os04g51190; LOC_Os07g40820; LOC_Os04g51190; LOC_Os06g02560; **LOC_Os03g51970**; LOC_Os12g29980; LOC_Os02g53690; LOC_Os03g47140; LOC_Os11g35030; LOC_Os01g08560; LOC_Os04g56740; LOC_Os12g10180; LOC_Os06g19380; LOC_Os05g10580; LOC_Os10g32680; LOC_Os04g13100; LOC_Os04g24190
96	osa-miR3979-5p	LOC_Os10g42400; LOC_Os01g49180; LOC_Os01g36910; LOC_Os10g30150; LOC_Os02g41810; LOC_Os03g64415; LOC_Os04g12060; LOC_Os04g54474; LOC_Os06g13370; LOC_Os06g06750; LOC_Os12g01970; LOC_Os06g37570; LOC_Os12g40900; LOC_Os04g57630; LOC_Os07g45890; LOC_Os10g04316; LOC_Os09g12720; LOC_Os04g01840; LOC_Os04g09330; LOC_Os09g32150; LOC_Os04g27590; LOC_Os05g33320; LOC_Os02g42060; LOC_Os11g43570; LOC_Os03g25620
97	**osa-miR397a**	LOC_Os01g62490; LOC_Os05g38410; **LOC_Os05g38420**; LOC_Os11g48060; LOC_Os01g63200; LOC_Os03g16610; LOC_Os01g44330; LOC_Os07g31310; LOC_Os10g04900; LOC_Os01g62480; LOC_Os08g35210; LOC_Os10g03850; LOC_Os05g38390; LOC_Os02g01710; LOC_Os03g31730
98	**osa-miR397b**	LOC_Os01g62490; LOC_Os05g38410; **LOC_Os05g38420**; LOC_Os11g48060; LOC_Os01g63200; LOC_Os12g15680; LOC_Os01g63180; LOC_Os03g16610; LOC_Os01g44330; LOC_Os02g01030; LOC_Os09g27950; LOC_Os07g31310; LOC_Os10g04900; LOC_Os08g35210
99	osa-miR399c	LOC_Os05g48390; LOC_Os05g45350; LOC_Os04g33860; LOC_Os06g19470
100	osa-miR399d	LOC_Os05g48390; LOC_Os05g45350; LOC_Os04g33860
101	osa-miR399i	LOC_Os02g13960; LOC_Os05g48390; LOC_Os05g45350
102	osa-miR399k	LOC_Os05g45350; LOC_Os05g48390; LOC_Os05g10310; LOC_Os07g14650 LOC_Os07g14650
103	**osa-miR444a**	**LOC_Os02g49840;** LOC_Os08g33488; LOC_Os02g36924; *LOC_Os04g38780*; LOC_Os02g17500; LOC_Os08g33479; LOC_Os03g62780; LOC_Os11g37100; *LOC_Os08g06510*
104	osa-miR444b	LOC_Os04g38780; LOC_Os02g36924; LOC_Os08g33488; LOC_Os02g49840; LOC_Os08g06510; LOC_Os08g33479; LOC_Os01g44990; LOC_Os10g02920; LOC_Os04g51350; LOC_Os11g42880; LOC_Os07g01250; LOC_Os02g01940; LOC_Os02g05410
105	osa-miR529b	*LOC_Os08g39890*; LOC_Os09g31438; LOC_Os01g69830; LOC_Os04g51830 *LOC_Os09g32944*; LOC_Os08g41940; LOC_Os08g34820; LOC_Os09g07300; LOC_Os05g04150; LOC_Os05g04150; LOC_Os05g14010; LOC_Os12g08760; LOC_Os01g65780; LOC_Os02g07720; LOC_Os04g33360; LOC_Os05g07090; LOC_Os05g07090; LOC_Os03g57900; LOC_Os02g03620; LOC_Os04g28420; LOC_Os04g28420; LOC_Os12g38110; LOC_Os02g38480; LOC_Os09g25900; LOC_Os03g10080; LOC_Os02g35910; LOC_Os01g60650; LOC_Os07g25484; LOC_Os06g28840; LOC_Os02g53200; LOC_Os02g53200
106	osa-miR530-5p	LOC_Os01g56780; LOC_Os07g16600; LOC_Os01g16180; LOC_Os01g16180; LOC_Os01g16180; LOC_Os01g16180; LOC_Os12g44340; LOC_Os03g63950; LOC_Os03g63950; LOC_Os11g47980; LOC_Os06g16600; LOC_Os04g47840;

		LOC_Os10g29450; LOC_Os04g51400; LOC_Os04g51400; LOC_Os04g51400; LOC_Os04g51400; LOC_Os03g18540; LOC_Os03g18540; LOC_Os03g52840; LOC_Os03g52840; LOC_Os03g52840; LOC_Os01g44430
107	osa-miR5337a	LOC_Os03g14590; LOC_Os09g31514; LOC_Os12g36870; LOC_Os05g50980; LOC_Os03g06139
108	osa-miR5337b	LOC_Os03g14590; LOC_Os09g31514; LOC_Os12g36870; LOC_Os05g50980; LOC_Os03g06139
109	osa-miR5338	LOC_Os12g38590; LOC_Os05g26040; LOC_Os03g58490; LOC_Os03g58490; LOC_Os05g29974; LOC_Os05g03000; LOC_Os05g03000
110	osa-miR5508	LOC_Os10g29180; LOC_Os03g16960; LOC_Os07g23220
111	osa-miR1428d	LOC_Os07g43460; LOC_Os01g01670; LOC_Os01g04720; LOC_Os01g04720
112	osa-miR1428e-3p	LOC_Os03g17980; LOC_Os07g43460; LOC_Os12g07900; LOC_Os11g06050; LOC_Os06g39600
113	osa-miR1428e-5p	LOC_Os01g66180; LOC_Os01g66180; LOC_Os08g05470; LOC_Os02g33850; LOC_Os11g42900
114	osa-miR1440a	LOC_Os07g27450; LOC_Os07g46080; LOC_Os07g46060; LOC_Os08g40615; LOC_Os07g45950; LOC_Os07g45760; LOC_Os07g45550; LOC_Os07g46030; LOC_Os07g45560; LOC_Os07g45560; LOC_Os04g30930; LOC_Os01g64810
115	osa-miR1440b	LOC_Os10g38040; LOC_Os03g63150; LOC_Os03g63150; LOC_Os04g28780; LOC_Os03g58230; LOC_Os03g44810; LOC_Os01g19140
116	osa-miR1441	LOC_Os05g03440; LOC_Os11g36620
117	osa-miR167a-3p	LOC_Os03g58260; LOC_Os03g58260; LOC_Os10g03230; LOC_Os04g58100; LOC_Os07g18840; LOC_Os04g58720; LOC_Os03g51090
118	osa-miR167a-5p	LOC_Os07g29820; LOC_Os01g63290; LOC_Os01g63290; LOC_Os10g37590; LOC_Os09g37890; LOC_Os06g03830; LOC_Os06g03830; LOC_Os07g49320; LOC_Os05g19050; LOC_Os04g18010
119	osa-miR1846d-3p	LOC_Os09g26760
120	osa-miR1855	LOC_Os01g14860; LOC_Os01g14860; LOC_Os01g14860; LOC_Os01g14860; LOC_Os05g08370; LOC_Os01g46610
121	osa-miR1871	LOC_Os06g19610; LOC_Os05g02350; LOC_Os02g27350; LOC_Os12g10470; LOC_Os07g26440; LOC_Os07g26440; LOC_Os07g26440; LOC_Os09g27480; LOC_Os12g07110; LOC_Os12g07110; LOC_Os05g16490; LOC_Os01g28300; LOC_Os02g50340; LOC_Os05g45330; LOC_Os07g45120; LOC_Os08g42530; LOC_Os06g06310; LOC_Os12g12190; LOC_Os08g23280; LOC_Os02g21320; LOC_Os06g17500; LOC_Os10g35160; LOC_Os05g28170
122	osa-miR1882g	LOC_Os03g02010; LOC_Os04g26490; LOC_Os03g18264; LOC_Os07g09580 LOC_Os11g35810; LOC_Os07g09580
123	osa-miR1882h	LOC_Os03g02010; LOC_Os04g26490; LOC_Os03g18264; LOC_Os07g09580; LOC_Os11g35810; LOC_Os07g09580
124	osa-miR2091-5p	LOC_Os12g42420; LOC_Os05g41010; LOC_Os01g49580; LOC_Os01g37980; LOC_Os05g24150; LOC_Os04g41460; LOC_Os04g10720
125	osa-miR2099-3p	LOC_Os02g54340
126	osa-miR2099-5p	LOC_Os03g30890; LOC_Os11g09510; LOC_Os03g30890; LOC_Os03g10750; LOC_Os05g06320; LOC_Os05g06320; LOC_Os07g10440; LOC_Os04g52361;

		LOC_Os02g27674; LOC_Os02g27674; LOC_Os11g05394; LOC_Os11g05394
127	osa-miR2864.1	LOC_Os09g07154; LOC_Os03g64290; LOC_Os11g06540; LOC_Os04g23760; LOC_Os03g11734; LOC_Os04g23720; LOC_Os04g06290; LOC_Os10g33484; LOC_Os02g50040; LOC_Os01g37310; LOC_Os03g55040; LOC_Os07g03040; LOC_Os07g03040; LOC_Os05g48790; LOC_Os05g48790; LOC_Os05g48790; LOC_Os02g05930; LOC_Os05g48790; LOC_Os03g55130; LOC_Os08g06130; LOC_Os05g31920; LOC_Os05g31920; LOC_Os04g32620; LOC_Os04g39260; LOC_Os12g36440; LOC_Os04g39260; LOC_Os04g39260; LOC_Os07g41600; LOC_Os02g47150; LOC_Os06g51250; LOC_Os06g10230; LOC_Os04g47320; LOC_Os02g46990; LOC_Os01g70330; LOC_Os11g41880; LOC_Os10g17790
128	osa-miR2864.2	LOC_Os02g16270; LOC_Os02g16270; LOC_Os01g55880; LOC_Os01g15540; LOC_Os02g36770; LOC_Os01g21820; LOC_Os05g25974; LOC_Os05g25974; LOC_Os01g56900; LOC_Os06g10290; LOC_Os09g34847; LOC_Os04g34360; LOC_Os09g34847; LOC_Os04g34410; LOC_Os02g54720; LOC_Os04g36830; LOC_Os03g64030; LOC_Os12g10340; LOC_Os06g36090
129	osa-miR2880	LOC_Os08g37360; LOC_Os03g40920; LOC_Os01g63830; LOC_Os04g58390
130	osa-miR2907a	LOC_Os05g18660; LOC_Os03g49990
131	osa-miR2923	LOC_Os01g72290; LOC_Os03g57460; LOC_Os01g13350; LOC_Os01g13350; LOC_Os01g48210; LOC_Os02g16490; LOC_Os04g49000; LOC_Os11g45190; LOC_Os01g70790; LOC_Os11g45130; LOC_Os01g64780; LOC_Os04g42480; LOC_Os04g54970; LOC_Os06g46670; LOC_Os07g48630; LOC_Os05g35010
132	osa-miR2926	LOC_Os01g13350; LOC_Os01g13350; LOC_Os04g52640; LOC_Os07g01340; LOC_Os01g16460; LOC_Os04g01570; LOC_Os12g41490; LOC_Os05g03640; LOC_Os04g56580; LOC_Os08g07740; LOC_Os10g38170; LOC_Os04g41560; LOC_Os11g18660; LOC_Os10g42490; LOC_Os10g42490; LOC_Os07g41580; LOC_Os08g20570; LOC_Os08g20570
133	osa-miR2930	LOC_Os06g14780; LOC_Os07g17070; LOC_Os02g48150; LOC_Os03g58700; LOC_Os11g35720; LOC_Os02g21770; LOC_Os03g54220; LOC_Os04g30430; LOC_Os02g49970; LOC_Os03g19820
134	osa-miR395m	LOC_Os03g09940; LOC_Os03g53230; LOC_Os03g09930; LOC_Os06g46480; LOC_Os11g44580; LOC_Os10g42439; LOC_Os05g29800
135	osa-miR395n	LOC_Os03g09940; LOC_Os03g53230; LOC_Os03g09930; LOC_Os06g46480; LOC_Os11g44580; LOC_Os10g42439; LOC_Os05g29800
136	osa-miR395o	LOC_Os03g53230; LOC_Os03g09940; LOC_Os03g07200; LOC_Os06g46480; LOC_Os03g09930; LOC_Os05g49890; LOC_Os02g01740; LOC_Os03g21250; LOC_Os07g38300
137	osa-miR395p	LOC_Os03g09940; LOC_Os03g53230; LOC_Os03g09930; LOC_Os06g46480; LOC_Os11g44580; LOC_Os10g42439; LOC_Os05g29800
138	osa-miR395q	LOC_Os03g09940; LOC_Os03g53230; LOC_Os03g09930; LOC_Os03g19750; LOC_Os06g46480; LOC_Os11g44580; LOC_Os10g42439; LOC_Os05g29800
139	osa-miR395r	LOC_Os03g09940; LOC_Os03g53230; LOC_Os03g09930; LOC_Os06g46480; LOC_Os11g44580; LOC_Os10g42439; LOC_Os05g29800
140	osa-miR395s	LOC_Os03g09940; LOC_Os03g53230; LOC_Os03g09930; LOC_Os06g46480; LOC_Os11g44580; LOC_Os10g42439; LOC_Os05g29800

141	osa-miR398a	LOC_Os07g46990;	LOC_Os01g68770;	LOC_Os11g27220	
142	osa-miR415	LOC_Os12g42280;	LOC_Os05g02520;	LOC_Os02g18320;	LOC_Os03g02514;
		LOC_Os03g17920;	LOC_Os04g46440;	LOC_Os06g02000;	LOC_Os03g61990;
		LOC_Os02g26810;	LOC_Os03g61990;	LOC_Os03g10290;	LOC_Os04g38630;
		LOC_Os02g01560;	LOC_Os02g56540;	LOC_Os01g72490;	LOC_Os06g51240;
		LOC_Os07g12810;	LOC_Os10g35690;	LOC_Os12g40890;	LOC_Os12g40560;
		LOC_Os05g37330			
143	osa-miR439a	LOC_Os12g11340			
144	osa-miR439c	LOC_Os12g11340			
145	osa-miR439e	LOC_Os12g11340			
146	osa-miR810b.1	LOC_Os01g65986;	LOC_Os02g03930		
147	osa-miR818a	LOC_Os07g40450;	LOC_Os09g36320;	LOC_Os03g15350;	LOC_Os01g63880;
		LOC_Os06g11500;	LOC_Os03g48010;	LOC_Os12g16350;	LOC_Os06g39330;
		LOC_Os03g63370;	LOC_Os02g14290;	LOC_Os02g10210;	LOC_Os01g63050;
		LOC_Os11g47160;	LOC_Os12g16290;	LOC_Os09g10710;	LOC_Os04g39160;
		LOC_Os06g05530;	LOC_Os01g12820;	LOC_Os03g49126;	LOC_Os08g29760;
		LOC_Os01g18850;	LOC_Os06g38210;	LOC_Os05g03610;	LOC_Os05g43570;
		LOC_Os01g41240;	LOC_Os01g51890;	LOC_Os02g43560;	LOC_Os09g37020;
		LOC_Os06g48060;	LOC_Os03g47960;	LOC_Os05g46490;	LOC_Os04g57154;
		LOC_Os09g07320;	LOC_Os08g37700;	LOC_Os10g42100;	LOC_Os08g37700;
		LOC_Os06g17950;	LOC_Os08g41080;	LOC_Os01g15970;	LOC_Os07g05145;
		LOC_Os01g72370;	LOC_Os01g57630;	LOC_Os07g48370;	LOC_Os03g53600;
		LOC_Os05g39540;	LOC_Os01g01720;	LOC_Os11g12060;	LOC_Os06g45890;
		LOC_Os03g25945			
148	osa-miR820b	LOC_Os03g02010;	LOC_Os11g13650;	LOC_Os11g03310;	LOC_Os05g00996;
		LOC_Os10g42196			
149	osa-miR821a	LOC_Os12g16350;	LOC_Os02g38340;	LOC_Os12g14840;	LOC_Os10g27274;
		LOC_Os02g32110;	LOC_Os05g34750;	LOC_Os06g48510;	LOC_Os04g40860
150	osa-miR821b	LOC_Os12g16350;	LOC_Os02g38340;	LOC_Os12g14840;	LOC_Os10g27274;
		LOC_Os02g32110;	LOC_Os05g34750;	LOC_Os06g48510;	LOC_Os04g40860

Note: The underline one was confirmed by degradome sequencing (Zhou et al., 2010; Li et al., 2010), while the bold one was validated by both degradome sequencing and experiment.

Zhou, M., Gu, L.F., Li, P.C., Song, X.W., Wei, L.Y., Chen, Z.Y. (2010). Degradome sequencing reveals endogenous small RNA targets in rice (*Oryza sativa* L. ssp. indica). Front. Biol. **5**(1): 67-90.

Li, Y.F., Zheng, Y., Quaye, C.A., Zhang, L., Saini, A., Jagadeeswaran, G., Axtell, M.J., Zhang, W.X., Sunkar, R. (2010). Transcriptome wide identification of microRNA targets in rice. Plant J. **62**, 742-759.

Supplementary Table S4 Details of reported miRNAs and their targets that is crucial for yield in rice.

miRNA	targets	Annotation	Strategy	effect	Reference
osa-miR156	LOC_Os08g39890	*OsSPL14*	Point mutation in *OsSPL14*	Reduce tiller,increase panicle branches	Jiao et al.,2010 Miura et al., 2010
osa-miR393a	LOC_Os05g05800	*OsTIR1*	Overexpression of miR393	Promote tillering	Xie et al., 2012
	LOC_Os04g32460	*OsAFB2*		Promote tillering	Xie et al., 2012
osa-miR397	LOC_Os05g38420	*Laccase*	Overexpression of miR397	Promote panicle branches, increase grain size	Zhang et al., 2013
osa-miR396b	LOC_Os03g51970	GRF4/6	Overexpression and MIM396	Modulate auxiliary branches, grain size	Gao et al., 2015 Duan et al., 2015
osa-miR172	LOC_Os05g03040		Overexpression and MIM172	Panicle	Wang et al., 2015
	LOC_Os07g13170			Panicle	Wang et al., 2015
osa-miR444a	LOC_Os02g49840	OsMADS57	Overexpression of miR444a	Suppressed tillering	Guo et al., 2013
osa-miR159a	LOC_Os01g59660	MYB		Panicle	Peng et al., 2014
osa-miR164e	LOC_Os03g47310	NAC		Grain filling	Peng et al., 2014
osa-miR1861	LOC_Os01g63810			Grain filling	Peng et al., 2014
osa-miR160a	LOC_Os06g47150		Northern blot	Grain filling	Lan et al., 2012
osa-miR167a	LOC_Os04g57610	ARF		Flag leaf	Xu et al., 2014
osa-miR171a	LOC_Os04g46860		Northern blot	Grain filling	Lan et al., 2012

Jiao, Y.Q., Wang, Y.H., Xue, D.W., Wang, J., Yan, M.X., Liu, G.F., Dong, G.J., Zeng, D.L., Lu, Z.F., Zhu, X.D., Qian, Q., Li, J.Y. (2010). Regulation of OsSPL14 by OsmiR156 defines ideal plant architecture in rice. Nat Genet. **6**(42) : 541-544.

Zhang, Y.C., Yu, Y., Wang C.Y., Li, Z.Y., Liu, Q., Xu, J, Liao, J.Y., Wang, X.J., Qu, L.H., Chen, F., et al. (2013). Overexpression of microRNA OsmiR397 improves rice yield by increasing grain size and promoting panicle branching. Nat Biotech. **31**: 848-852.

Guo, S., Xu, Y., Liu, H., Mao Z., Zhang, C., Ma, Y., Zhang, Q., Meng, Z., Chong, K. (2013). The interaction between OsMADS57 and OsTB1 modulates rice tillering via DWARF14. Nat Commun. **4**: 1566.

Gao, F., Wang, K., Liu, Y., Chen, Y.P., Chen, P., Shi, Z.Y., Luo, J., Jiang, D.Q., Fan, F.F., Zhu, Y.G., Li, S.Q. (2015). Blocking miR396 increases rice yield by shaping inflorescence architecture. Nat Plants **2**: 15196.

Duan, P.G., Ni, S., Wang, J.M., Zhang, B.L., Xu, R., Wang, Y.X., Chen, H.Q., Zhu, X.D., Li, Y.H. (2015). Regulation of OsGRF4 by OsmiR396 controls grain size and yield in rice. Nat Plants, **2**: 15203.

Miura, K., Ikeda,M., Matsubara, A., Song, X.J., Ito, M., Asano, K., Matsuoka, M., Kitano, H., Ashikari, M. (2010). OsSPL14 promotes panicle branching and higher grain productivity in rice. Nat Genet. **42**: 545-549.

Xie, K., Wang, R., Ou, X., Fang, Z., Tian, C., Duan, J., Wang, Y., Zhang, M. (2012). OsTIR1 and OsAFB2 downregulation via OsmiR393 overexpression leads to more tillers, early flowering and less tolerance to salt and drought in rice. PloS ONE **7**: e30039.

Wang, L., Sun, S.Y., Jin, J.Y., Fu, D.B., Yang, X.F., Weng, X.Y., Xu, C.G., Li, X.H., Xiao, J.H., and Zhang, Q.F. (2015). Coordinated regulation of vegetative and reproductive branching in rice. Proc Natl Acad Sci USA. **112**: 15504-15509.

Lan, Y., Su, N., Shen, Y., Zhang, R.Z., Wu, F.Q., Cheng, Z.J., Wang, J.L., Zhang, X., Guo, X.P., Lei, C.L., et al. (2012). Identification of novel miRNAs and miRNA expression profiling during grain development in indica rice. BMC Genomics **13**: 264.

Peng, T., Sun, H., Qiao, M., Zhao, Y., Du, Y., Zhang, J., Li, J., Tang, G., Zhao, Q. (2014). Differentially expressed microRNA cohorts in seed development may contribute to poor grain filling of inferior spikelets in rice. BMC Plant Biol. **14**:196.

Xu, X., Bai, H., Liu, C., Chen, E., Chen, Q., Zhuang, J., Shen, B. (2014). Genome-wide analysis of microRNAs and their target genes related to leaf senescence of rice. PloS one **9**(12): e114313.

Supplementary Table S5 Primers for qRT-PCR used in this study.

**Gene**	**Sequences (5'-3')**
miR393a-F	GTCGCTCCAAAGGGATCGC
miR393a-RT	GTCGTATCCAGTGCAGGGTCCGAGGTATTCGCACTGGATACGACGATCAA
miR171a-F	GCTCTCTGATTGAGCCGCG
miR171a-RT	GTCGTATCCAGTGCAGGGTCCGAGGTATTCGCACTGGATACGACGATATT
Universal-R	GTGCAGGGTCCGAGGT
U6-F	CAACGGATATCTCGGCTCT
U6-R	CAACTTGCGTTCAAAGACTC
